# Integrating biological and environmental data to solve key scientific and societal challenges

**DOI:** 10.1093/biosci/biaf150

**Published:** 2025-10-15

**Authors:** David M Kunkel, Brooke L Long-Fox, Cameron Pittman, Julia Portmann, Matthew Sheik, John M Bates, Andrew Bentley, Dori L Contreras, Elizabeth R Ellwood, Michael W Lomas, Anna K Monfils, William E Moser, Gil Nelson, Sinlan Poo, Barbara Thiers, Gregory J Watkins-Colwell, Michael S Webster, Breda M Zimkus, Jyotsna L Pandey

**Affiliations:** Department of Biology, Oklahoma State University, Stillwater, Oklahoma, United States; MorphoBank, Phoenix Bioinformatics, Newark, California, Department of Chemistry, Biology, Health Science, South Dakota School of Mines and Technology, Rapid City, South Dakota, United States; Denver Museum of Nature and Science, Denver, Colorado, United States; Yale School of the Environment, Yale University, New Haven, Connecticut, United States; Denver Botanic Gardens, Denver, Colorado, United States; Negaunee Integrative Research Center, Field Museum, Chicago, Illinois, United States; Biodiversity Institute, University of Kansas, Lawrence, Kansas, United States; Perot Museum of Nature and Science, Dallas, Texas, United States; iDigBio, University of Florida, Gainesville, Florida, United States; National Center for Marine Algae and Microbiota, Bigelow Laboratory for Ocean Sciences, East Boothbay, Maine, United States; Department of Biology, Central Michigan University, Mount Pleasant, Michigan, United States; National Museum of Natural History, Smithsonian Institution, Suitland, Maryland, United States; iDigBio, Florida Museum of Natural History, University of Florida, Gainesville, Florida, United States; Department of Conservation and Research, Memphis Zoological Society, Memphis, Tennessee, United States; New York Botanical Garden, Bronx, New York, United States; Yale Peabody Museum, New Haven, Connecticut, United States; Cornell Lab of Ornithology, Cornell University, Ithaca, New York, United States; Museum of Comparative Zoology, Harvard University, Cambridge, Massachusetts, United States; American Institute of Biological Sciences, Herndon, Virginia, United States

**Keywords:** best practices and standards, biodiversity collections, cross‑disciplinary collaboration, data integration, stakeholder engagement

## Abstract

Biodiversity collections in the United States hold over a billion specimens and are essential to understanding the history of life on Earth, as well as patterns of biodiversity in response to environmental change. Each specimen is linked by metadata to an organism's name and the place and time of its collection. Extensive data have been collected on Earth's geology, hydrology, climate, and organisms—past and present—but the data remain largely fragmented. We report in the present article on community discussions to develop a roadmap and identify action items for the Building an Integrated, Open, Findable, Accessible, Interoperable, and Reusable (BIOFAIR) Data Network, directly linking the various types of biological and environmental data. The roadmap is organized into five themes: stocktaking and gap analysis, technological capacity building, best practices, education and training, and community building. Together, these themes chart a path from initial resource inventories and skill building to infrastructure development, cross‑disciplinary collaboration, and the establishment of FAIR‑compliant workflows and governance.

Addressing the complex societal and scientific challenges of our time—from biodiversity loss and climate change to emerging public health threats—demands a collaborative and interdisciplinary approach to science. This necessitates integrating large volumes of diverse data, expertise, and perspectives. Although the amount of digital, biological, and environmental data has dramatically increased in recent decades, data resources largely remain siloed. Providing open data and ensuring clear attribution have been shown to drive greater equity, innovation, and reuse in open science (Buschbom et al. 2025). The crucial next step is intentional data integration, creating accessible and interoperable data resources that support transformative, cross-domain research, training, and public engagement.

Biodiversity collections, including over a billion specimens in the United States, offer unparalleled information for understanding evolution, biological processes, and biodiversity responses to environmental change (NASEM 2020). Their value is greatly amplified when linked to associated data such as genomic, phenotypic, and environmental information (Lendemer et al. 2020), but realizing this potential requires a coordinated, collaborative effort.

To this end, the Biodiversity Collections Network (BCoN), in collaboration with the American Institute of Biological Sciences (AIBS), spearheaded the “Building an Integrated, Open, Findable, Accessible, Interoperable, and Reusable (BIOFAIR) Data Network” project, funded by the US National Science Foundation (NSF Division of Biological Infrastructure [DBI] award no. 2303588). The aim of this initiative was to engage diverse stakeholders through a series of domain-focused virtual listening sessions and an interdisciplinary workshop, fostering collaborations to advance an integrated network of biological and environmental data.

This effort directly builds on the Extended Specimen Network vision (Thiers et al. 2019, Lendemer et al. 2020). The Extended Specimen Network connects the extended specimen concept—physical specimens linked to their associated information (e.g., genetic, phenotypic, environmental data, media, audio recordings)—with the essential actions needed to build, support, use, and sustain a BIOFAIR network. These actions include continued collection, sustained digitization, advancements in physical and cyberinfrastructure, and comprehensive education and training.

Expanding on the Extended Specimen Network vision and the complementary international digital extended specimen effort (Hardisty et al. 2022), the BIOFAIR Data Network project responded to a clear community need: to unite data producers, managers, and users across disciplines. The goal was to explore common objectives and define a shared vision for an integrated biological and environmental data network. This effort is especially timely amid urgent threats, including accelerating biodiversity loss, climate change, and emerging zoonotic diseases, which underscore the importance of accessible, well-curated biodiversity data. By uniting stakeholders across biological and environmental sciences, this project aims to strengthen and align data integration efforts, broaden community engagement, and foster a data-enabled, inclusive scientific workforce for the twenty-first century.

An integrated data network, as was envisioned by the BIOFAIR Data Network project, could enable transformative research across biology, ecology, public health, and environmental science. By linking biological specimens with diverse environmental, genomic, and other digital data, researchers could investigate complex questions about species adaptation, biodiversity loss, and ecosystem function. This infrastructure could support forecasting of changes in biodiversity, predicting the distribution and spread of invasive species, and evaluating the impact of land use or conservation policies. It could also inform public health by providing valuable insight into how global biodiversity affects disease dynamics. Ultimately, such a data network could support the creation of a digital twin (Blair 2021, de Koning et al. 2023, Schigel 2024) of Earth's ecosystems, allowing scientists to perform virtual experiments and generate actionable insights for a rapidly changing world.

The 2-year BIOFAIR Data Network project (2024–2025) leveraged interdisciplinary expertise to catalyze cross-domain dialogue, identify shared biological and environmental data needs and goals, and define actionable next steps toward the development of a data network that supports digital integration and drives scientific innovation. Six virtual listening sessions were focused on federal agencies, genetics and genomics data, One Health, climate and environmental data, ecological data, and biodiversity informatics, and engaged 199 stakeholders representing 142 organizations. The participants explored current barriers to data discovery, standardization, equity, training, and sustainability, and generated a set of community-driven recommendations. During these listening sessions, six critical community-identified needs emerged: the needs to enhance data availability; to improve capacity for data integration; to establish long-term sustainable funding models and streamlined data infrastructure; to build a robust training infrastructure for data stewardship; to ensure equitable and ethical data access; and to establish best practices for data use, curation, and citation (figure 1).

**Figure 1.**
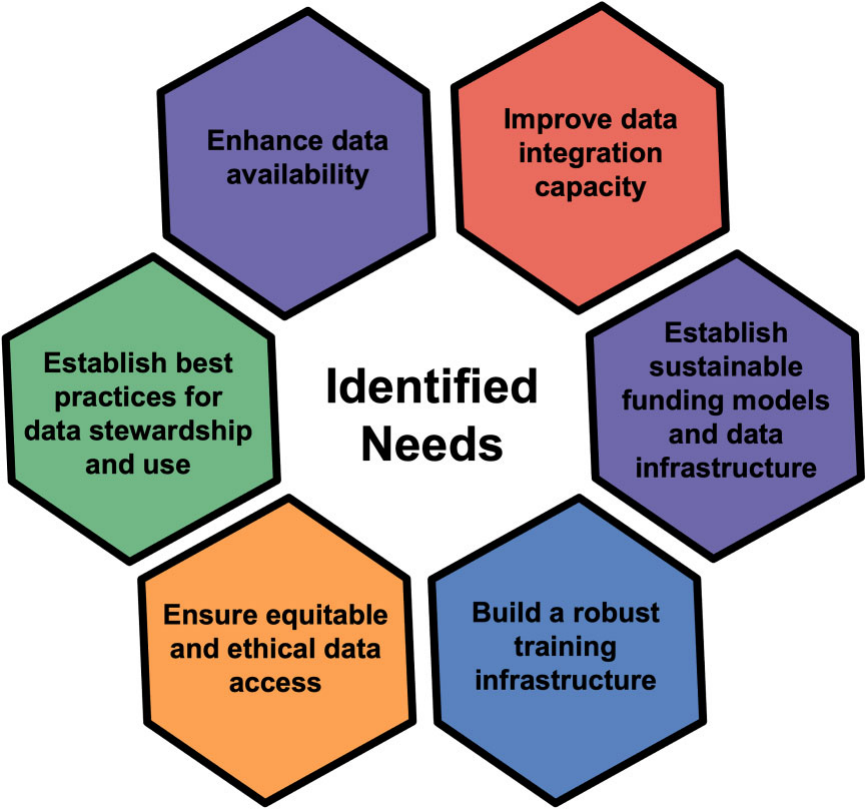
Illustration of the BIOFAIR Data Network project's six community-identified needs for building an integrated biological and environmental data network. These needs emerged across the six listening sessions focused on federal agencies, genetics and genomics data, One Health, climate and environmental data, ecological data, and biodiversity informatics. Credit: Brooke Long-Fox.

A subsequent virtual workshop was convened to further explore the community needs identified during the listening sessions. A subset of the listening session participants interested in further discussion were invited to attend, and speakers and additional topical experts were included to further inform discussions of both the technological and social dimensions of an integrated data network, including sustained cross-disciplinary collaboration, robust infrastructure, ethical and inclusive data governance, equitable access, and investment in human capital, technological innovation, and capacity building. The workshop engaged 75 stakeholders, collectively representing 110 organizations.

The discussions highlighted several key considerations for building this integrated network encompassing both technological and social dimensions. The technological challenges include developing and implementing robust infrastructure and methodologies for effective data integration, which are crucial for making biodiversity data more accessible and interoperable. Closely tied to these technological challenges, the social challenges relate to issues of ethical responsibility, collaboration, and sustainability. The speakers emphasized embedding ethical considerations and community engagement in data sharing, highlighting the need for infrastructure investment, local data control, and culturally responsive data governance, particularly for researchers in the Global South. Strong, cross-cultural, interdisciplinary communities are vital to fostering effective scientific cooperation. Ethical data use, especially involving Indigenous knowledge, requires governance models that prioritize relational accountability and recognize traditional knowledge systems as valid data sources. The discussions also underscored the importance of incorporating CARE (for *collective benefit, authority to control, responsibility, ethics*) data principles (Carroll et al. 2020) and developing flexible data-sharing frameworks that support data sovereignty and local context labeling. Sustainability, both environmental and the equitable distribution of research benefits, remains a central theme across all discussions.

During the workshop, the participants collaboratively refined an actionable and adaptable roadmap that would provide direction toward an integrated data network. The roadmap addressed each of the six community-identified needs, highlighting key milestones toward success and generating over 150 sociotechnical strategies that were subsequently synthesized into the priority cross-cutting themes described below.

## Priority cross-cutting themes

**Figure 2.**
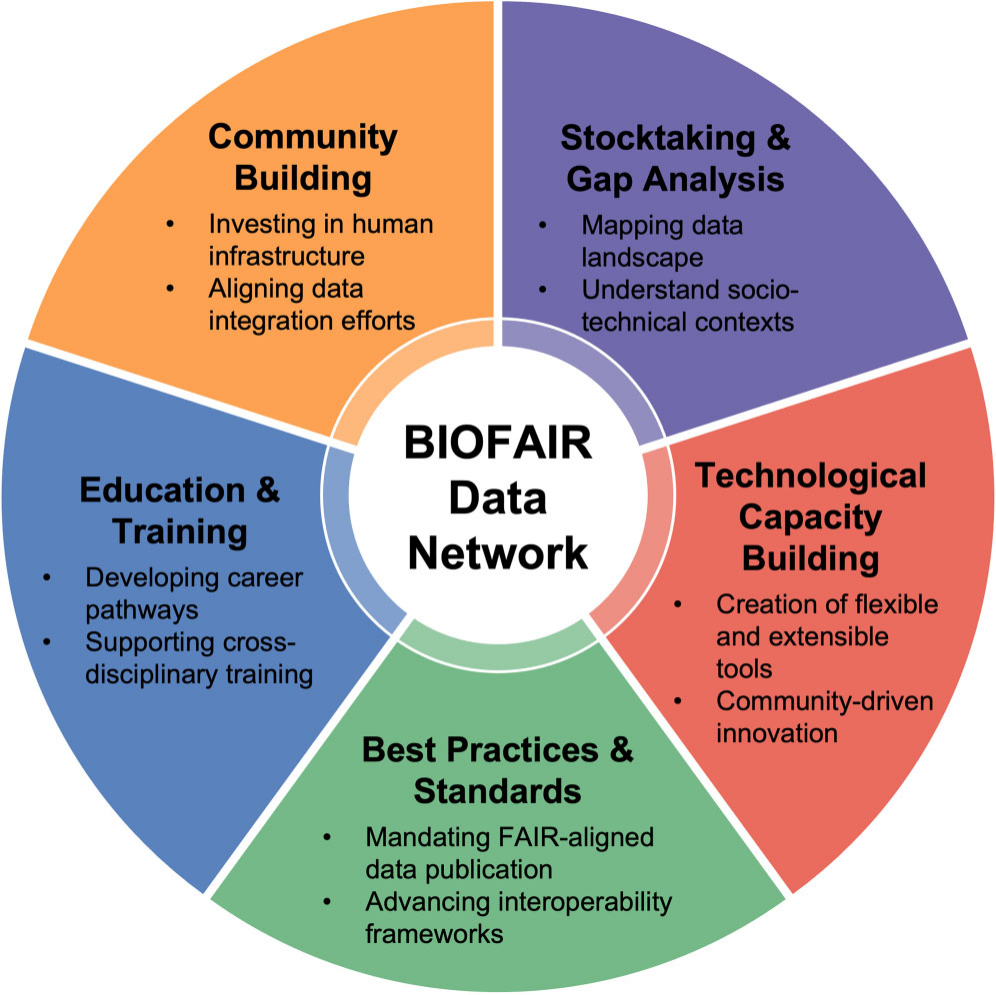
Illustration of the cross-cutting themes identified in the community-developed roadmap toward an open and integrated data network. Credit: Cameron Pittman and Matthew Sheik.

Five cross-cutting themes emerged from the roadmap discussions: the needs for taking stock of existing and missing resources, technological capacity building, developing a set of best practices and standards, education and training, and community building (figure 2). These themes align with the listening sessions and workshop presentations, reflecting a shared vision for an integrated biological and environmental data network that builds on existing assets; that is supported by inclusive community structures; that is strengthened through education and technological capacity; and that is guided by clear, ethical, and interoperable standards. In the present article, we present summaries of the strategies included in each theme, with more detailed information included in the refined roadmap tables (supplemental material).

### Stocktaking and gap analysis

Just as a researcher studies an ecosystem by investigating its components, the BIOFAIR discussions emphasized identifying the interconnectivity, redundancy, and discipline-specific variations across data domains regarding their social and technological landscapes. This includes quantifying data entities (e.g., data types, repositories, aggregators) and compiling associated controlled vocabulary standards.

For example, a starting point could be mapping the interconnectedness of Darwin Core (Darwin Core Task Group 2009), Ecological Metadata Language (Jones et al. 2019), the International Organization for Standardization's geographic information metadata schema (ISO 2014, 2019, 2023), and other data models. Doing so provides opportunities to coalesce redundant definitions and delineate gaps for data types not currently mapped.

The workshop discussions also called for collaborations across siloed databases, a concern echoed in recent NSF initiatives, such as the Biology Integration Institutes (NSF 23-511) and Integrative Research in Biology (NSF 21-622) programs. By compiling information related to currently disparate data infrastructures, we can foster collaboration through identifying unrepresented data types, sustainability models, and current infrastructure overlaps. This will include advancing data access and integration tools, providing storage for contemporary data types (e.g., acoustic and visual data), and adopting effective sustainability models for long-term support.

Understanding the use of existing tools in creating, transforming, and integrating data is key to developing future flexible and extensible data tools for integrating current and contemporary data types. A recent report from the National Academies of Sciences, Engineering, and Medicine on continental-scale biology examined analytical and sampling tools, networks, and synthesis centers to identify current gaps and limitations across temporal and spatial scales. The authors of the study concluded that investment in new sensor modalities to improve data collection, next-generation digitization of biodiversity collections, and integrating spatially and temporally variable data is necessary for continental-scale biological studies (NASEM 2025).

Finally, our discussions emphasized the need to assemble existing best practices for every step in the data lifecycle, from data creation to data storage. An anthology of best practices documentation across disciplines could draw inspiration from NASA's SciXplorer (https://scixplorer.org/), a literature-based, open digital information system focusing on space science research. The compilation of best practices into one platform would enable analyses identifying and addressing gaps in data management workflows, as well as incorporate previously excluded viewpoints from underrepresented voices and indigenous knowledge holders.

### Technological capacity building

Supporting the collection, storage, and management of data requires both physical and digital infrastructure, integrated into a cohesive network. Organizations such as the Distributed System of Scientific Collections (DiSSCo) and the Global Biodiversity Information Facility (GBIF) are taking steps toward this by creating networks “to mobilize the data, skills, and technologies needed to make comprehensive biodiversity information freely available” (GBIF 2023). This effort reflects the need for technological capacity building, a cross-cutting theme emphasized throughout the workshop and presentations. The overarching goal is to strategically invest in integrated, interoperable, and inclusive technological infrastructure that enhances data availability, accessibility, maintenance, equity, and usability.

To achieve this, several key priorities must be addressed. These include the development and adoption of common metadata templates, data models, and identifier resolution systems to advance standardization and interoperability. These recommendations mirror the Earth Science Information Partners Physical Samples Curation Cluster's “Publishing Open Research Using Physical Samples” guidelines (Damerow et al. 2025), which include spreadsheet templates, application programming interface (API) submissions, and parent–child personal identifiable data hierarchies for subsamples. Such measures, supported by the use of shared APIs, collaboration with software engineers, and the application of AI or machine-learning tools, will strengthen data provenance, integration, and adherence to FAIR principles (data that are findable, accessible, interoperable, and reusable; Wilkinson et al. 2016), while reducing duplication across repositories, content management systems, and domains.

Repositories should be incentivized to establish formal connections with adjacent repositories, fostering interoperable systems and shared interests in data integration. Collaboration with and among funding agencies is also essential to invest in resilient, FAIR-aligned cyberinfrastructure that supports scalable data integration, replication, and access regardless of location. By promoting these partnerships, resources can be more inclusively distributed, enabling underresourced institutions to access critical hardware, software, and IT support. Cyberinfrastructure must also be made resilient to technical, economic, and other disruptions. This can be achieved through mechanisms for data mirroring, synchronization, and relocation and by ensuring access independent of hosting location, supported through collaborative funding projects and small institutional awards aimed at widespread adoption. In addition, there is a need to develop flexible tools and integration strategies that accommodate emerging technologies and future data types, ensuring the longevity and adaptability of data systems.

To support community-driven innovation, infrastructure that incorporates data stewardship standards should be codeveloped with journals, repositories, and data scientists. Repository interconnectivity and shared stewardship responsibilities should be incentivized through dedicated funding and recognition mechanisms. Ultimately, the success of such infrastructure depends equally on the strength and cohesion of the community that supports and sustains it.

### Best practices and standards

Establishing FAIR-aligned data integration across repositories, tools, institutions, geographies, and scientific disciplines begins with a coordinated, cross-sector effort to define, adopt, and continuously evolve best practices and standards. Central to this effort is the expectation that authors publish their data concurrently with research outputs through community-recognized portals. Damerow and colleagues (2025) provided a field-tested author guide for assigning, citing, and updating persistent sample identifiers at every stage from field collection through data publication, ensuring long-term traceability and reproducibility. This process requires not only technical infrastructure but also a commitment to data sharing, standards optimization, integration procedures, equity-focused practices, and long-term flexibility.

Recent work has shown that the noncopyrightability of underlying data, when paired with robust attribution mechanisms, significantly enhances equity, innovation, and reuse (Buschbom et al. 2025). Equally critical is the optimization of shared standards and interoperability frameworks. Controlled vocabularies, such as Darwin Core, identifier systems (i.e., DOIs), and metadata schemas must be expanded, harmonized, and regularly maintained to support seamless data exchange and reduce semantic mismatches. For example, the Biodiversity Information Standards Working Group (TDWG) has extensive repositories of best practice metadata reporting and already maintains some unique identifier standards and protocols (e.g., Pereira et al. 2009, GUID 2011). Building on this repository would be beneficial, particularly to address another key point raised, which is to improve digital asset maintenance by institutions.

Managing digital assets is only sustainable if each is uniquely identified. Whether standards are maintained by TDWG or other groups, institutions should take the lead in ensuring that their repositories follow standardized protocols to allow for improved data accessibility.

Shared editorial tools and repository workflows should be developed to automate identifier assignment, facilitate the automatic linking of data sets to research outputs, and streamline deposition into centralized, FAIR-compliant systems that will help authors meet publisher requirements and make data submission easier and more consistent. To embed data integration as a recognized professional norm, publishers, funding agencies, conferences, and academic institutions must collaborate to integrate FAIR-aligned practices across the entire research lifecycle. Adoption of these standards should be tied to professional recognition systems, such as grant eligibility, tenure and promotion criteria, certification programs, and other career advancement pathways. This alignment incentivizes high-quality data stewardship as a core component of scholarly excellence.

A truly FAIR data ecosystem must also be equitable, secure, and accessible. Establishing best practices for equity, security, and access requires a coordinated framework grounded in the CARE principles (Carroll et al. 2020, 2021, 2022a, 2022b, Jennings et al. 2023). Clear guidelines must be developed for data access, protection, and use, including privacy-respecting tiered access models ranging from fully open portals to secure data visitation environments. Infrastructure support for regions with limited connectivity (e.g., lightweight APIs, offline data packages, and regional mirror sites) is essential to ensure inclusive participation. A unified integration approach that balances openness with responsibility should embed embargo and redaction options, apply local context labels to honor community-defined permissions and data sovereignty, and implement usage logging and automated attribution to track downstream reuse and ensure proper credit for data providers.

Finally, best practices and standards must evolve through formalized, iterative processes. Regular community review workshops, public comment periods, and version-controlled, openly accessible standards repositories allow integration guidelines to adapt in response to technological advancements and shifting ethical landscapes. The development of automated quality-monitoring tools (e.g., those that track vocabulary alignment, verify identifier resolution, and support data annotation, citation, and cross-platform recognition) will ensure that integration standards remain robust, responsive, and inclusive for a diverse and evolving research community.

### Education and training

Strengthening education and training resources is integral to developing a culture of FAIR, open data. The roadmap contributors emphasized the importance of building FAIR-data training to increase data stewardship capacity through institutional support, community integration, and clear standards. Currently, many educational programs do not cover FAIR and open data principles in adequate detail, although some progress is being made (e.g., Shanahan et al. 2021).

In addition to strengthening undergraduate and graduate curricula, the participants emphasized that organizations should prioritize professional development and continuing education modules for staff to provide updated training as FAIR principles evolve and new data tools are developed. Educational resources would also benefit from modules focused on data and metadata standards, interoperability, and stewardship. Some relevant training materials may be available through the ELIXIR training e-Support System portal, which aggregates varied life sciences–related training modules and workshops. As well as expanded training resources, more effort is needed to strengthen peer-to-peer networks for data resource sharing.

One relevant example is the Museums and Emerging Pathogens in the Americas (MEPA) ECHO group; they have built a community of practice around pathogen data sharing and education for working beyond the standard research scientist community (Colella et al. 2021). MEPA has accomplished this through cross-disciplinary training, regular virtual meetings, and sharing best practices for biorepository standardization, serving as a model of what a BIOFAIR network could look like.

Another key topic that emerged was improving training resources for data accessibility and stewardship, including in the use of unique, accessible, and cross-disciplinary identifiers. The Australian Research Data Commons has taken one approach to addressing this issue by developing a freely available “FAIR Data 101” module hosted on GitHub targeted at professionals working with data (Liffers et al. 2021). In addition, some data stewardship certification programs already exist (e.g., SAS Global Certification Program) and are useful starting points. However, we envision data stewardship programs specific to the environmental and biological sciences. This could be accomplished by the creation of society or institution-based accreditation programs and certifications rather than those hosted by private companies. Integral to these trainings would be clear guidance on metadata standards for improving data interoperability.

Creating biodiversity-centered data integration and training resources is essential as we move forward with developing a BIOFAIR data network, and we hope that biodiversity institutions can take the lead.

### Community building

Core to laying the foundation for the development of a BIOFAIR data network is the need to build an inclusive, collaborative, and sustainable community of data providers, managers, and users that can integrate across technical, educational, and policy boundaries to support collective data sharing. Our data communities are often siloed within their respective disciplines, which creates difficulties for understanding our current data landscape and being able to coordinate efforts to address some of our greatest societal and scientific challenges. By engaging with and fostering collaboration among data users, providers, and technical experts across data domains, we can begin to increase the capacity for data integration that aligns with FAIR data principles across data systems. For example, LAGOS-US, a national database of lakes, required extensive and ongoing efforts to consolidate data, which had been collected and organized in disparate ways and without consistent unique identifiers, into a comparable framework. Improving synergistic team collaboration was essential to doing this, and led to the development of new training resources (e.g., Cheruvelil and Soranno 2018, Oliver et al. 2018).

In building a core community across data domains, we also can align data integration efforts that have already been undertaken across various data communities to support ongoing and future research. These integration efforts can be supported further through the development of accessible guidance and funding mechanisms, as well as intentional efforts to distribute technical expertise. Doing so is of paramount importance to address inequities in access to data, training, and expertise between the Global North and Global South (Mwampamba et al. 2020, McAlvay et al. 2021). In addition, we need to be mindful of not only supporting our existing workforce but also advocating for an increased capacity for long-term data stewardship. This can be accomplished by increasing the visibility of careers for future generations, sharing data use success stories, and training in FAIR data skills and competencies. In doing so, we ultimately invest in developing a greater workforce capacity that supports the data manager and mobilization specialist positions necessary to carry forward and maintain data integration efforts into the foreseeable future and beyond. When considering training in ethical data stewardship, it is essential that we, as a scientific community, include stewards of traditional knowledge in the discussion, and acknowledge and incorporate traditional knowledge systems when investigating biodiversity questions (Wildcat 2009). By instilling in the next generation of data stewards the importance of including traditional knowledge in our integrated data systems, we can develop a workforce that is not only trained in how to manage and keep data, but also mindful of the communities contributing to the data that they are managing.

Finally, these efforts need to be supported across multiple platforms, from publishers to funding agencies and other supporting institutions. Some efforts are already being made in this domain to create more inclusive communities across data domains. A key NSF-funded initiative moving the community in this direction is the Environmental Science Innovation and Inclusion Lab (ESIIL), which seeks to create an environment in which scientists can work across scientific domains to address complex interdisciplinary scientific questions (https://esiil.org/). In addition, the International Partners for the Digital Extended Specimen (IPDES) acts as a group of representatives from a broad spectrum of organizations, including GBIF, iDigBio, AIBS, BCoN, Atlas of Living Australia, DiSSCo, and others, seeking to facilitate coordination of a global network of stakeholders to achieve the vision of the digital extended specimen. Their community allows for deeper and more frequent communication and collaboration across the globe. In building communities that advocate for interdisciplinary collaboration around the world, such as ESIIL and IPDES, we can effectively establish a long-term support network that promotes FAIR data standards and maintains the necessary infrastructure to make the BIOFAIR Data Network a reality.

## Next steps

The success of the envisaged BIOFAIR Data Network requires coordinated and increased investment in both social and technical infrastructure, sustained collaboration, and a shared commitment to equity, openness, and innovation. Building on momentum generated through community listening sessions and interdisciplinary workshops, the BIOFAIR initiative is positioned to catalyze transformative change in how biological and environmental data are integrated, accessed, and reused. Realizing this vision will depend on formalizing collaborative pathways that bridge disciplinary silos and center both technical interoperability and social responsibility.

A crucial next step is the creation of expert-led, funded working groups that include both technical developers and social scientists. These collaborative groups would conduct a comprehensive stocktaking of data types, tools, and controlled vocabularies across disciplines to identify gaps, redundancies, and opportunities for alignment. Understanding how integration tools are used and where they need improvement will guide the design of more responsive, user-centered solutions. These foundational works will aid education professionals in creating tailored learning resources to address community training needs, skill gaps, and FAIR-related competencies for those developing best practices. Ultimately, we emphasize the need for coordination across social and technological disciplines to achieve an open, FAIR biodiversity data network.

Another critical next step involves aligning with and learning from complementary efforts such as the Bouchout Declaration and the Disentis Roadmap (2025), ESIIL, the Integrated Postsecondary Education Data System, and the Zoo–Museum Network. Coordinated engagement with these and other initiatives offers a model for establishing cross-sector partnerships, codeveloping shared infrastructure, and advancing policy and funding mechanisms that promote open, ethical, and inclusive science. To build a truly integrated network, these efforts must prioritize meaningful participation from underrepresented communities, support local data stewardship, and incorporate diverse knowledge systems, including Indigenous and traditional ecological knowledge.

The BIOFAIR Data Network project is both a vision and a blueprint. Realizing its potential will require intentional, sustained action: mobilizing resources, cultivating partnerships, and building the trust and capacity necessary to support a twenty-first-century data ecosystem. Dedicated investment is essential to ensure the development of resilient infrastructure, the cultivation of a diverse and skilled workforce, and the establishment of inclusive governance models that prioritize ethical data use and long-term stewardship. We invite all who engage with biological and environmental data to progress this vision forward through identifying and obtaining funding sources, building domestic and international collaborations, and investing time and resources into our identified first steps toward developing an integrated data network that follows FAIR and CARE data principles.

## Supplementary Material

biaf150_Supplemental_Files

## Author Biography

David M. Kunkel is affiliated with the Department of Biology at Oklahoma State University, in Stillwater, Oklahoma, in the United States. Brooke L. Long-Fox is affiliated with MorphoBank, at Phoenix Bioinformatics, in Newark, California, and with the Department of Chemistry, Biology, and Health Science at the South Dakota School of Mines and Technology, in Rapid City, South Dakota, in the United States. Cameron Pittman is affiliated with the Denver Museum of Nature and Science, in Denver, Colorado, in the United States. Julia Portmann is affiliated with the Yale School of the Environment, at Yale University, in New Haven, Connecticut, in the United States. Matthew Sheik is affiliated with Denver Botanic Gardens, in Denver, Colorado, in the United States. John M. Bates is affiliated with the Negaunee Integrative Research Center, at the Field Museum, in Chicago, Illinois, in the United States. Andrew Bentley is affiliated with the Biodiversity Institute at the University of Kansas, in Lawrence, Kansas, in the United States. Dori L. Contreras is affiliated with the Perot Museum of Nature and Science, in Dallas, Texas, in the United States. Elizabeth R. Ellwood is affiliated with iDigBio, at the University of Florida, in Gainesville, Florida, in the United States. Michael W. Lomas is affiliated with the National Center for Marine Algae and Microbiota, at the Bigelow Laboratory for Ocean Sciences, in East Boothbay, Maine, in the United States. Anna K. Monfils is affiliated with the Department of Biology at Central Michigan University, in Mount Pleasant, Michigan, in the United States. William E. Moser is affiliated with the National Museum of Natural History, at the Smithsonian Institution, in Suitland, Maryland, in the United States. Gil Nelson is affiliated with the iDigBio, at the Florida Museum of Natural History, at the University of Florida, in Gainesville, Florida, in the United States. Sinlan Poo is affiliated with the Department of Conservation and Research at the Memphis Zoological Society, in Memphis, Tennessee, in the United States. Barbara Thiers is affiliated with the New York Botanical Garden, in the Bronx, New York, in the United States. Gregory J. Watkins-Colwell is affiliated with the Yale Peabody Museum, in New Haven, Connecticut, in the United States. Michael S. Webster is affiliated with the Cornell Lab of Ornithology, at Cornell University, in Ithaca, New York, in the United States. Breda M. Zimkus is affiliated with the Museum of Comparative Zoology at Harvard University, in Cambridge, Massachusetts, in the United States. Jyotsna L. Pandey (jpandey@aibs.org) is affiliated with the American Institute of Biological Sciences, in Herndon, Virginia, in the United States.
