# Supplementary material for: Integrating biological and environmental data to solve key scientific and societal challenges
Source: Bioscience. 2025 Oct 15;76(1):13–20. doi: 10.1093/biosci/biaf150 (PMC12771510; doi:10.1093/biosci/biaf150)
Supplement: biaf150_Supplemental_Files [file biaf150_supplemental_files.zip › BIOFAIR Data Network Project Final Report.pdf]

BIOFAIR DATA NETWORK

# Building an Integrated, Open, Findable, Accessible, Interoperable, and Reusable Data Network

## Final Report

July 28, 2025

BIODIVERSITY COLLECTIONS NETWORK

AMERICAN INSTITUTE OF BIOLOGICAL SCIENCES

NSF

## Table of Contents


Preface....... 3

Chapter 1: Introduction....... 4

- Need for an Integrated Data Network....... 4
- Overview of Activities....... 5
- Community Engagement....... 6

Chapter 2: Listening Sessions Reveal Broad Consensus....... 8

- Community-Identified Needs and Recommendations for Addressing Data Integration and Implementation of a BIOFAIR Data Network....... 8
- Future Directions....... 12

Chapter 3: Current Data Integration Landscape: A Summary of Presentations....... 13

- Program....... 13
- Challenges....... 14
- Future directions....... 15

Chapter 4: Developing the Roadmap Towards a BIOFAIR Data Network....... 16

- Cross-Cutting Themes in the Roadmap....... 17
- Future directions....... 20

Chapter 5: Enabling the Future of Data Integration....... 22

- The BIOFAIR Data Network....... 22
- Other related and on-going projects....... 23
- Collections-based Biodiversity Data....... 24
- Where do we go from here?....... 25

References....... 26

Authorship of this Document....... 28

Appendices (Separate Document)....... 29

- A: List of Participants....... 29
- B: BIOFAIR Data Network Workshop Agenda....... 29
- C: Stakeholders Master List....... 29
- D: What could the BIOFAIR Network enable?....... 29
- E: Refined Roadmap Tables....... 29
- F: Enhanced AI Summary....... 29

## Preface

*“Nature is the domain of liberty,’ Humboldt said, because nature’s balance was created by diversity which might in turn be taken as a blueprint for political and moral truth. Everything, from the most unassuming moss or insect to elephants or towering oak trees, had its role, and together they made the whole. Humankind was just one small part. Nature itself was a republic of freedom.”*

**— Andrea Wulf, The Invention of Nature: Alexander von Humboldt's New World**

*“It really boils down to this: that all life is interrelated. We are all caught in an inescapable network of mutuality, tied into a single garment of destiny. Whatever affects one directly, affects all indirectly.”*

**— Martin Luther King Jr., Christmas sermon of 1967, Ebenezer Baptist Church, Atlanta**

Since at least the time of Alexander von Humboldt (1756–1859), humans have been recording information about the vast array of organisms with whom we share this planet, their interrelationships, and the physical characteristics of the habitats in which they live. As of the 21st century, we have accumulated a breadth of diverse data, much of it now in digital form, to ask questions about fundamental processes on Earth, and to do what we can still do to preserve the web of life that fascinates and sustains us. We not only have data, but also powerful means for analyzing those data to test hypotheses, including hypotheses about the growing impact of human behavior on our environment and the organisms with which we share (or have shared) our home. It is the responsibility of those who create, manage, and use biological and environmental data to ensure that we maximize investments in data capture and preservation made over the past several centuries and  ensure that the tools for and the results from the analysis of these data are accessible to everyone. In this way, we do what we can to ensure that Martin Luther King’s “network of mutuality tied into a single garment of destiny,” which so aptly describes the interdependencies of life, is made durable by threads woven from comprehensive, verifiable data about our natural world, and that it fits everyone.

## Chapter 1: Introduction

The Biodiversity Collections Network (BCoN), in collaboration with the American Institute of Biological Sciences (AIBS), conducted a series of domain-focused virtual listening sessions followed by an interdisciplinary, collaborative workshop in 2024–2025 as part of the National Science Foundation (NSF) funded Building an Integrated, Open, Findable, Accessible, Interoperable, and Reusable (BIOFAIR) Data Network project (DBI Award No. 2303588). These events were purposefully designed to engage a broad set of stakeholders with the goal of fostering novel, timely collaborations to advance the development of an integrated network of biological and environmental data—including genomic, ecological, climate, geological, biodiversity, behavioral, and human health data.

These discussions built upon the Extended Specimen Network (ESN) vision proposed in BCoN's 2019 report, Extending U.S. Biodiversity Collections to Promote Research and Education (1, 2). The report connected the Extended Specimen concept—physical specimens (living and preserved) and their associated digital information, including genetic, phenotypic, and environmental data, and media, such as audio recordings of soundscapes and focal species—with a suite of supporting actions needed to derive, support, utilize, and sustain the ESN. This includes continued collection, sustained digitization, advancement of both physical and cyberinfrastructure, and education and training of our workforce and user communities. Key findings from the 2019 BCoN report, such as the call for specimen management plans for newly funded research (3) and the creation of a collections action center (4), were later echoed in the National Academies of Science, Engineering, and Medicine (NASEM) report on biological collections (5), and incorporated into the bipartisan CHIPS and Science Act of 2022 (Public Law No: 117-167).

By leveraging interdisciplinary expertise within the collections community and beyond, BCoN and AIBS brought together stakeholders across biological and environmental data domains to explore opportunities for building a collaborative partnership. The objective was to catalyze cross-domain dialogue, identify shared biodiversity data needs and goals, and define actionable next steps toward an integrative and expanded data network that supports digital integration and drives scientific research and innovation.

### Need for an Integrated Data Network

Addressing major societal and scientific challenges—now and in the future—including complex environmental issues affecting biodiversity, public health, and agriculture, requires an inclusive, interdisciplinary approach to science that integrates diverse data, expertise, and perspectives. While recent decades have seen a dramatic increase in digital biodiversity, ecological, and environmental data, these resources remain largely fragmented and/or un-standardized. The critical next step is intentional data integration—creating accessible, interoperable resources that support transformative, cross-domain research, training, and public engagement.

Biodiversity collections, which maintain more than one billion U.S. specimens representing the history of life on Earth, offer unparalleled information for understanding evolution, biological

processes, and responses to environmental change. The value of these specimens is amplified when effectively linked to derivative and associated data (e.g., genomic, phenotypic, environmental), but realizing their full potential demands a coordinated and collaborative effort.

Building on the vision of the ESN, and the subsequent international Digital Extended Specimen effort (6), this project responded to a clear community need: to bring together data producers, managers, and users across disciplines to explore common goals and define a shared vision for an integrated biological and environmental data network. This effort is especially timely amid urgent threats, including biodiversity loss, climate change, and emerging zoonotic disease, which underscore the importance of accessible, well-curated biodiversity data. By uniting stakeholders across biodiversity, ecological, and environmental sciences, the project aims to strengthen data integration, broaden community engagement, and support a data-enabled, inclusive scientific workforce for the 21st century.

**Overview of Activities**

With the aim of engaging the next generation of biodiversity professionals in this collaborative effort, the BIOFAIR Data Network project Steering Committee kicked-off the effort with the recruitment of emerging and early career professionals to join the leadership team through an open call to the biodiversity research community. Six individuals (Abeyrathna, Kunkel, Long-Fox, Pittman, Portmann, and Sheik) were selected and invited to join the Steering Committee to facilitate the planned community engagements.

In spring 2024, the expanded steering committee participated in two workshops to boost the team's effectiveness and inclusivity. The first workshop focused on principles of team science, effective meeting facilitation, and leadership skills. The second workshop focused on creating and facilitating inclusive spaces and elevating diverse voices in STEM.

Following these training sessions, the steering committee formed six Planning Committees, each tasked with organizing a virtual listening session. The listening sessions focused on six broad data domains: federal agencies, genetic and genomic data, climate and environmental data, ecological data, One Health, and biodiversity informatics. Each planning committee identified and invited key representatives from their respective domain to assist with the organization of the listening session. Invited key domain representatives (KDRs) played a critical role in identifying important stakeholders to invite from within their disciplines and helped determine appropriate speakers and points of discussion during each session. Listening sessions were held in summer of 2024, culminating in a cross-cutting summary report released in October 2024 (Chapter 2). Upon completion of the report, the steering committee held a public webinar on November 13, 2024 to discuss the report and gather additional feedback.

Insights from listening sessions, the summative report, and public webinars informed the February 13, 2025 final workshop. This virtual event brought together a diverse and interdisciplinary group of stakeholders to develop recommendations and a roadmap towards a Findable, Accessible, Interoperable, and Reusable (FAIR), open, integrated biological and environmental data network. The steering committee synthesized these discussions into a draft

final report, which was shared with listening session and workshop participants for feedback before its finalization.

| Timeline of Activities | 2023 | | 2024 | | | | 2025 | |
|---|---|---|---|---|---|---|---|---|
| Activity | Q3 | Q4 | Q1 | Q2 | Q3 | Q4 | Q1 | Q2 |
| Meetings of the steering committee | ■ | ■ | ■ | ■ | ■ | ■ | ■ | ■ |
| Recruitment of emerging and early career professionals | ■ | | | | | | | |
| Completion of training workshops on team and inclusive science | | | ■ | | | | | |
| Finalize listening session planning committees | | | ■ | | | | | |
| Planning and participant recruitment for listening sessions | | | ■ | ■ | | | | |
| Six domain-focused listening sessions | | | | ■ | ■ | | | |
| Asynchronous online input gathering on listening session summary reports | | | | | ■ | | | |
| Finalize and share cross-cutting summary report from listening sessions | | | | | | ■ | | |
| Planning and participant recruitment for the workshop | | | | | | ■ | | |
| Final virtual workshop | | | | | | | ■ | |
| Preparation of final report, roadmap, and publication | | | | | | | ■ | ■ |
| Asynchronous online input gathering on final report and roadmap | | | | | | | | ■ |
| Webinar summarizing project outcomes | | | | | | | | ■ |
| Roadmap, final report, and publication finalized and publicized | | | | | | | | ■ |

**Community Engagement**
The BIOFAIR Data Network project engaged a vast community of biological and environmental data stakeholders from various disciplines. The listening sessions brought together 199 experts, not including steering committee members, collectively representing 142 projects or institutions (Appendix A).

Subsequently, the final interdisciplinary workshop convened an expansive group of 75 stakeholders, including 19 steering committee members and a subset of attendees from the listening sessions, collectively representing or affiliated with 110 organizations, initiatives, and agencies, to discuss a path forward towards a FAIR, open, integrated biological and environmental data network (Appendix A). Figure 1.1 shows the distribution of workshop

participants based on their self-identified roles (researcher, educator, informatician, administrator, etc.) and how they interact with data (producer, consumer, manager, publisher, etc.). Many respondents identified multiple roles and interactions, highlighting the diverse expertise and perspectives represented in the workshop discussions.

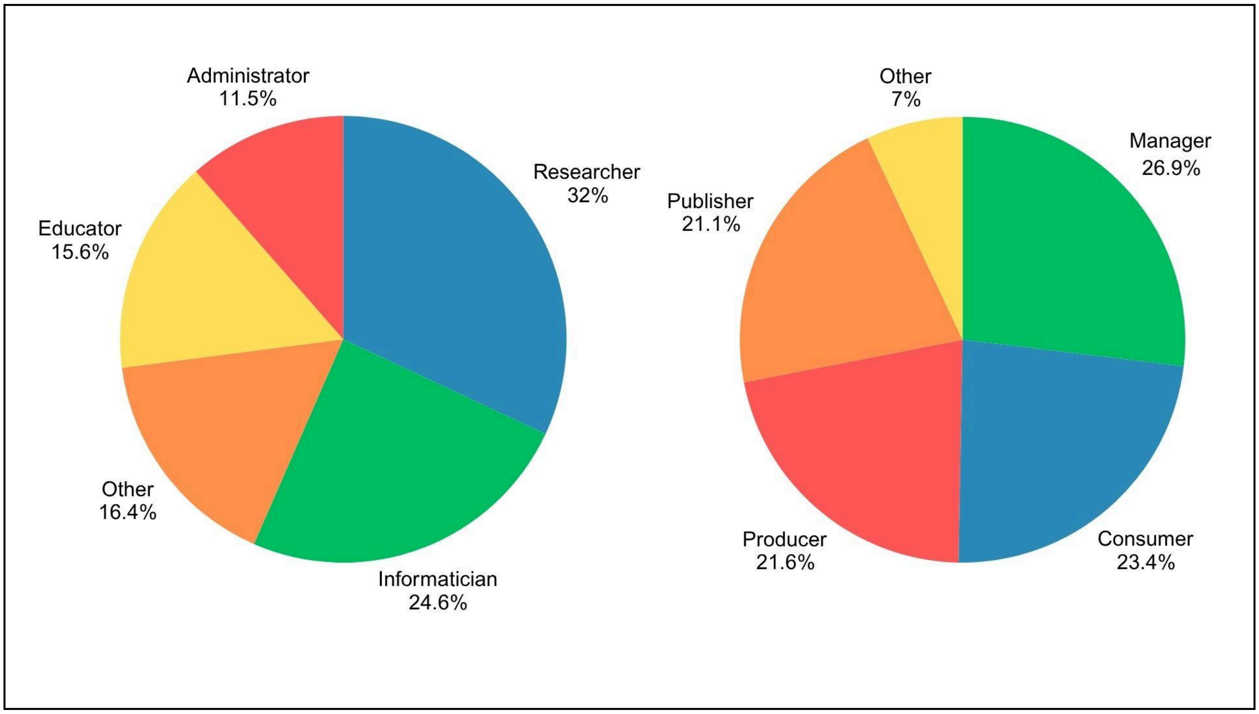


*Figure 1.1. Left: Distribution of workshop participants based on self-identified roles; Right: Participant distribution based on how they work with data.*

The following chapters summarize the discussions and findings from the virtual listening sessions and the final interdisciplinary workshop, and elaborates on next steps forward.

## Chapter 2: Listening Sessions Reveal Broad Consensus

The listening sessions marked the first phase of the BIOFAIR Data Network project. Enhancing the value of biological specimens through durable linkages to derivative and associated data will require not only robust technical infrastructure, but also the establishment of social norms and data-sharing agreements that are not yet fully in place. These sessions served as an initial step toward building a broad, cross-disciplinary data community to support the integration and sharing of biological and environmental data.

These themed sessions brought together a novel configuration of early career, emerging and established professionals, from a wide range of backgrounds, expertise, and perspectives for six, two-hour discussions led by steering committee members and key domain representatives. The six themes were: Federal Agency Data (June 14), Genetic and Genomic Data (June 26), One Health Data (2 July), Ecological Data (July 12), Climate and Environmental Data (July 2), and Biodiversity Informatics (August 26).

This chapter focuses on shared themes that emerged across the listening sessions. The wide range of perspectives shared during the listening sessions are documented in the individual session summaries accessible through the BIOFAIR Data Network website (7).

Listening session participants indicated strong support for the vision of a globally accessible data network that would serve the data needs of a broad range of potential users and help improve and sustain the individual data resources that constitute the network. However, participants recognized that significant barriers exist to the realization of this vision. Broadly categorized, these barriers include limitations to data discovery and access, inconsistent standardization, insufficient training, and insecure data sources. Many of the recommendations to overcome these barriers would benefit from the development of a collective impact model for FAIR biological and environmental data. A collective impact model is a structured approach to tackling complex social issues by bringing together different organizations to work toward a common goal (8). The perceived barriers and recommendations for overcoming them are described in detail below.

### Community-Identified Needs and Recommendations for Addressing Data Integration and Implementation of a BIOFAIR Data Network

#### *Address equity in data access*

Bandwidth and access issues in some regions, including low- and middle-income countries and rural and remote communities, prevent even data access and sharing. This means that we are not only missing data from climatically sensitive, biodiverse regions of the world, but also excluding the full participation of scientists in these regions. Participants across all six listening sessions assigned a high priority to addressing global inequities in the provision of, and access to, raw and synthesized data. Collectively, the recommendations outlined in this report aim to democratize data access. However, concerted efforts will be needed to ensure these recommendations are effective in diverse global contexts.

***Establish standards and protocols for ensuring ethical use of data***
Data access strategies must balance FAIR goals with CARE (Collective Benefit, Authority to Control, Responsibility, Ethics) data principles that provide necessary data protections. We must ensure that legal and ethical considerations (e.g., data sovereignty, attribution, and permissions surrounding traditional knowledge) are incorporated into models for data sharing and integration across different data jurisdictions. For example, researchers, institutions, and data publishers could engage with indigenous communities and incorporate mechanisms like Local Context labels and notices (9) to recognize and protect traditional knowledge associated with data. Database providers could explore options for data embargoes or redactions to comply with regulations and protect sensitive information, and the community could hold further discussions around third-party ownership of genetic sequence information to allow for editing and correction in perpetuity.

***Foster best practices***
We must collect data using established protocols that enable integration with other datasets and support reuse in research with differing objectives to those of the original study for which they were collected. Documentation of datasets should include the generation of complete and standardized metadata with details on data cleaning methods, analyses, and synthesized data storage, while clean or augmented data should be shared back to data providers. To help comply with such recommendations, resources need to be created to better describe data types and tools to integrate across domain-specific data types. A roadmap (or concept map) of data integration efforts (e.g., users, contributors, repositories, data aggregators) would guide future data integration. A system of credit or recognition for submitting data and metadata that adheres to best practices might also incentivize researchers to make data publicly available.

Data repositories and aggregators should develop mechanisms for data curation and annotation by institutions and communities without altering original data submissions, and provide clear guidance on how to cite material from their collection, including unique identifiers and linking protocols. Data users should cite data sources so that providers are aware of how their data are being used. Community and participatory science data and the complexities of these disciplines need to be included in discussions about biological data integration. Data repositories such as the Barcode of Life Data System (BOLD) (10), the Global Biodiversity Information Facility (GBIF) (11), and the International Nucleotide Sequence Database Collaboration (INSDC) (12) should continue to develop user-friendly tools and templates to facilitate standardized metadata submission by researchers and consider implementing translation layers among different data standards and formats. Ecological data communities, including the National Ecological Observatory Network (NEON) (13) and the Long Term Ecological Research Network (LTER (14), in conjunction with the biodiversity informatics community, need to continue developing and adopting data and research metadata standards, enabling harmonization of data across networks and interoperability of ecological data repositories with biodiversity informatics systems.

Journals, publishers, and their editorial board members should establish common standards for citing specimen (including associated tissues) and sequence data in publications, including supplemental materials, with specimen catalog numbers and sequence IDs, submitting authors, institutions, and usage details. Funding agencies should require that data and specimen management plans be submitted with research proposals that describe plans for data sharing, citation, and deposition in appropriate repositories, and provide guidance to grant applicants on how to develop and budget for such activities (15).

***Increase data availability***

We are still missing the data needed for a comprehensive network of biological and environmental data. Gaps in data identified by listening session participants include datasets that underlie research projects, especially small and focused datasets from individual researchers and monitoring programs, “non-standard” data types such as acoustic recordings, telemetry, and data gathered in participatory or community science projects. Despite recent massive efforts to digitize biological specimens, participants noted that information on current (as opposed to historical) species occurrence data and species absence data are still needed. In some cases, data may exist but are not shared due to concerns about cybersecurity, embargos for publication priority, and/or over-sampling of sensitive species. In addition, many providers restrict the use of their data by the private sector, even though the private sector is a major provider and user of environmental data; similarly, there are restrictions within the private sector to the wide sharing of their data and analyses.

Participants suggested that institutions, projects, and individuals holding data resources must continue to prioritize the digitization of these resources as well as continue to improve these resources through augmentation and standardization. Community leadership, perhaps in the form of a sustainably funded data action center, could address data access barriers as described above, and ensure that a biological and environmental data network is compatible with existing infrastructure projects, for example, the Distributed System of Scientific Collections (DiSSCo) (16), GBIF, and the Internet of Samples (iSamples) (17), and promotes interdisciplinary research, particularly incorporating aspects of the social sciences when human impacts are a major component of a research project.

***Improve data integration***

Currently, we do not have a common language, ontologies, or data models that are flexible enough to support a BIOFAIR Data Network. Some components of published biological data are underutilized because they have insufficient or non-standardized metadata. We need to expand resources that describe data types to facilitate integration across domain-specific data. Such a resource could take the form of a catalog of data resources that could be used to find needed datasets and also could be leveraged to determine gaps in current data availability. A gap analysis would reveal where most of our biodiversity information comes from, where it is utilized, and how the development of missing resources need to be prioritized.

Data integration would be facilitated by enhancements to the Darwin Core model (18) such as the Humboldt Core Extension for Ecological Inventories (19) designed to improve the integration

of ecological monitoring data (e.g., acoustic monitoring, camera trap, and animal movement data) and other extensions, including a single system of persistent, resolvable identifiers required for a Digital Extended Specimen architecture. Similarly, collaboration with emerging initiatives like the Biodiversity Digital Twin project (BioDT) (20) would be beneficial. A digital twin is a virtual representation of objects or systems designed to reflect physical entities accurately, and BioDT uses advanced simulations to tackle global biodiversity challenges, offering practical tools and insights for conservation and restoration. Differences in methodologies and scale of data represent another barrier to integration, and we need clarification regarding applicable standards and limitations of historical versus newly gathered data and a plan for each as it relates to data types to make these fully interoperable. Researchers, database providers, publishers, and funding agencies should explore the use of persistent identifiers such as Digital Object Identifiers (DOIs) for linking specimen data across different databases and repositories, while considering the funding challenges for low- and middle-income countries, by devising brokering services to provide the necessary linking infrastructure.

***Provide data competency training***
Participants in multiple sessions commented on the need for more robust education or training resources to ensure the effective use and maintenance of biological and environmental datasets. The quantity and breadth of interdisciplinary research are limited not only by data availability, but also by a lack of skills for appropriate and innovative data use by potential users. Participants called for the development of human resources that focus on data sharing and integration. Research communities should prioritize hiring data managers and provide training opportunities in data management skills as career advancement for current personnel. Educational institutions should prioritize data management and standards training for early career scientists. Improved user training and knowledge products are needed. Attribution metrics for data collection and curation must be established and broadly disseminated to highlight human contributions throughout the data lifecycle.

***Sustain and enhance existing data resources***
Many key data resources lack a plan for sustained funding. Data resources that underlie research analyses and conclusions must remain available so they can be used to reproduce this research. Therefore, a biological and environmental data community must explore sustainable funding and infrastructure models for long-term data preservation and access through increased engagement with public and private funding sources. We should also take steps to unify the landscape of multiple aggregators such as GBIF, the Global Genome Biodiversity Network (GGBN) (21), Integrated Digitized Biocollections (iDigBio) (22), the Ocean Biodiversity Information System (OBIS) (23), and VertNet (24) to provide a simpler, more user-friendly experience while also exploring more efficient publishing mechanisms to link specimens to citations, e.g., Biodiversity Literature Repository (25), CETAF e-publishing EJT (26) Pensoft ARPHA publishing tool (27). Advances in artificial intelligence, in particular the biodiversity-focused conversational interface iChatBio (28), allow users to query multiple aggregators such as GBIF, iDigBio, and the Biodiversity Heritage Library (BHL) (29) simultaneously, though further linkages are needed and planned to reach the scale of access necessary for addressing today’s research question.

**Future Directions**

Insights from these six listening sessions informed the development of a preliminary roadmap outline for the creation of an integrated, open, and FAIR data network. This outline served as a foundation for discussions during the subsequent workshop, part of the second phase of the BIOFAIR Data Network project described below. The goal of the final workshop, which engaged a subset of listening session participants, as well as additional relevant data community members, was to augment and refine the roadmap, identify key milestones, and suggest impactful use cases to guide the development of the integrated data network.

## Chapter 3: Current Data Integration Landscape: A Summary of Presentations

The BIOFAIR Data Network workshop agenda (Appendix B) included nine invited presentations across disciplines, institutions, and geographies to discuss data integration, biodiversity, and environmental research. Collectively, the presentations built on key themes identified in the listening sessions (Chapter 2), including the need for sustained cross-disciplinary collaboration, robust infrastructure, ethical and inclusive data governance, equitable access, and investment in human capital. The invited presentations underscored the need for technological innovation and capacity-building to address global environmental challenges. Many of the presentations highlighted global data-sharing initiatives and/or projects focused on fostering collaboration across disciplines, which provide models of success for the BIOFAIR Data Network project.

Interspersed between these presentations (workshop recording available at https://youtu.be/OmuxKpInr2; summary available in Appendix F), discussion sessions were held while workshop participants also developed a master stakeholder list of people and organizations that need to be involved in developing a BIOFAIR network (Appendix C), discussed previous efforts and associated challenges in data integration, and brainstormed possible applications of a realized BIOFAIR data network (Appendix D). A key component of the workshop was a group exercise to develop a roadmap for building a FAIR, open, and integrated biological and environmental data network, which is described in detail in Chapter 4.

### Program

The first session of the workshop was focused on the “Technological Aspects of BIOFAIR” and included four presentations:

- Kyle Copas (Communications Manager, Global Biodiversity Information Facility (GBIF)) presented on “Current data integration efforts at GBIF”, discussing the social challenges of data sharing and integration.
- Sharif Islam (Data Architect, Distributed System of Scientific Collections (DiSSCo)) highlighted the role of “Digital twin concept and examples, DiSSCo” in enhancing biodiversity data accessibility through the DiSSCo project.
- Jeannine Cavender-Bares (Director, Harvard University Herbaria) presented “A Vision for Continental-Scale Biology”, emphasizing the integration of biological research across multiple scales.
- Anand “Sunny” Narayanan (Lead Ambassador, NASA Science Explorer Digital Library (SciX)) introduced the “SciX - Science Explorer Digital Library Portal”, a cross-disciplinary digital library for open-access scientific literature.

The four presentations given during the second half of the workshop were focused on the “Social Aspects of BIOFAIR”:

- Kendra Spence Cheruvelil (Professor, Michigan State University) shared insights on “Building a community – Collective impact model” for effective interdisciplinary teams.
- Joseph Cook (Distinguished Professor and Curator, Museum of Southwestern Biology) spoke on ‘’Building the MEPA community” focused on the *Museums and Emerging*

*Pathogens in the Americas (*MEPA) project, which integrates museum biorepositories with biomedical research and response.
- Israel Borokini (Assistant Professor, Montana State University) addressed “Challenges towards building a global BIOFAIR data network”, particularly in the Global South
- Daniel Wildcat (Faculty, Haskell Indian Nations University) offered an “Indigenous perspective on data sharing” and ethical stewardship.

The closing presentation was given by James Macklin (Research Scientist, Agriculture and Agri-Food Canada) who discussed the “Genomic Adaptation and Resilience to Climate Change - GenARCC” project, highlighting the role of genomic tools in assessing climate change impacts on biodiversity and tying together many of the topics touched on by previous presenters.

**Challenges**

Speakers identified common challenges in data integration that stem from both technological and social barriers. While technological limitations exist, social and cultural factors often pose greater obstacles to effective data sharing and integration. Differences in access to digital infrastructure, funding, and expertise create global disparities, particularly between the Global North and Global South. Issues of data sovereignty underscore the need to respect Indigenous rights over data collection and governance, ensuring that research practices align with ethical and cultural considerations.

***Technological considerations***

Technical challenges involve the development and implementation of robust infrastructure and methodologies for effective data integration. Aligning digital specimens with FAIR principles is crucial for making biodiversity data more accessible, interoperable, and machine-readable. Presentations at the BIOFAIR workshop reinforced this priority, highlighting how technical innovation must be paired with thoughtful design and cross-domain coordination. These efforts illustrate how large-scale research depends on coordinated observational networks, standardized metadata models, and emerging tools such as artificial intelligence (AI) to study biodiversity across broad spatial and temporal scales. Digital platforms showcase how cross-disciplinary, open-access digital libraries and repositories can improve data accessibility and interoperability.

***Social considerations***

Speakers highlighted the importance of embedding ethical considerations and community engagement in data sharing. Social challenges in data integration revolve around ethical responsibility, collaboration, and sustainability. For example, there are challenges to data integration from the perspective of researchers in the Global South, emphasizing the need for infrastructure investment, local control of data, and culturally responsive data governance. The need for strong, interdisciplinary communities is vital for fostering effective scientific cooperation. Ethical data use, particularly concerning Indigenous knowledge, requires governance models that prioritize relational accountability over extractive research practices. One workshop presentation offered an Indigenous perspective, urging institutions to prioritize accountability and recognize traditional knowledge systems as equally valid sources of data. Their insights

echoed listening session discussions on the importance of incorporating CARE principles and developing flexible data-sharing frameworks that support data sovereignty and local context labeling. Sustainability, both in terms of environmental stewardship and equitable distribution of research benefits, remains a central theme across discussions.

**Future directions**

Moving forward, it will be critical to strengthen global data-sharing networks and advance the vision of an integrated biological and environmental data network by 1) building on existing infrastructures (e.g., GBIF, DiSSCo, etc.) to create improved networks of interoperable platforms that connect datasets across disciplines, 2) expanding interdisciplinary collaborations through new partnerships to co-design solutions, 3) refining data integration through good governance systems, made possible by technical advances such as AI that enable widespread and seamless automation, to ensure equitable and culturally-sensitive data governance and use, 4), advancing the Darwin Core schema to be more flexible to capture more data types (e.g., ecological networks), and linking with schemata from associated research areas, and 5) developing user-friendly tools and clear guidelines for data contributors to encourage broader participation from underrepresented research communities.

## Chapter 4: Developing the Roadmap Towards a BIOFAIR Data Network

The roadmapping exercise, conducted during the BIOFAIR Data Network workshop, was designed to facilitate a collaborative, actionable, adaptable, and community-informed roadmap for building a FAIR, open, and integrated biological and environmental data network. This was accomplished through the formation of a subcommittee that led the development of the roadmap exercise through iterative, focus-group-style pilots that included the entire steering committee for exercise revision. The core goal was to guide participants through a structured, participatory discussion aimed at generating key socio-technical strategies and milestones that contribute to addressing the six broad needs that emerged as priorities from the listening session discussions (summarized in the table below and described in Chapter 2).

| **Identified Needs** |
| --- |
| 1. Enhance the availability of biological and environmental data to support research and decision-making.<br>2. Improve capacity for data integration.<br>3. Establish sustainable funding models and streamlined infrastructure to ensure the long-term preservation, accessibility, and usability of key biological and environmental data resources.<br>4. Build a robust training infrastructure to equip researchers, data managers, and early career scientists with the skills needed for the effective use, sharing, and maintenance of biological and environmental datasets.<br>5. Develop mechanisms for addressing inequities in data access and policy to ensure the ethical use of data.<br>6. Incentivize researchers, database providers, and publishers to establish and adhere to best practices for data use, curation, and citation. |

The roadmapping exercise divided participants into six groups representing the six identified needs. Individuals were placed in groups based on a pre-workshop survey that provided participants with the opportunity to rank their interest in the six identified needs. Each group comprised at least seven participants and three steering committee member facilitators. Three milestones were provided to each group, derived from discussions from the 2024 listening sessions, and workshop participants were allowed to add additional milestones. Guided by BCoN facilitators, participants then discussed several SMART (specific, measurable, achievable, relevant, and time-bound) strategies to achieve each milestone. During these discussions, facilitators asked participants to also envision measures to assess progress, short- and long-term outcomes, and the resources needed for each conceived strategy. Ideas and discussions were captured in information tables in real-time by both BCoN facilitators and workshop participants.

The raw roadmap tables were cleaned up, organized, and refined for readability (Appendix E) after the workshop in order to develop a synthesized roadmap to guide near-term actions and a

long-term vision. This included distilling the substantial amount of information generated into a set of common themes that emerged across all six groups, which are further discussed below.

**Cross-Cutting Themes in the Roadmap**

Across the above six roadmapping groups, five cross-cutting, overarching themes emerged: the need for taking stock of existing and missing resources; technological capacity-building; developing a set of best practices and standards; education and training; and community building. These cross-cutting themes align with the listening sessions and workshop presentations, reflecting a shared vision for an integrated biological and environmental data network that builds on existing assets, is supported by inclusive community structures, is strengthened through education and technological capacity, and is guided by interoperable standards and ethical best practices. Here we present brief summaries of the strategies included in each theme, with more detailed information included in the refined roadmap tables (Appendix E). Of the five themes, stocktaking and gap analysis was seen as the first step critical for advancing the overall goals of the roadmap.

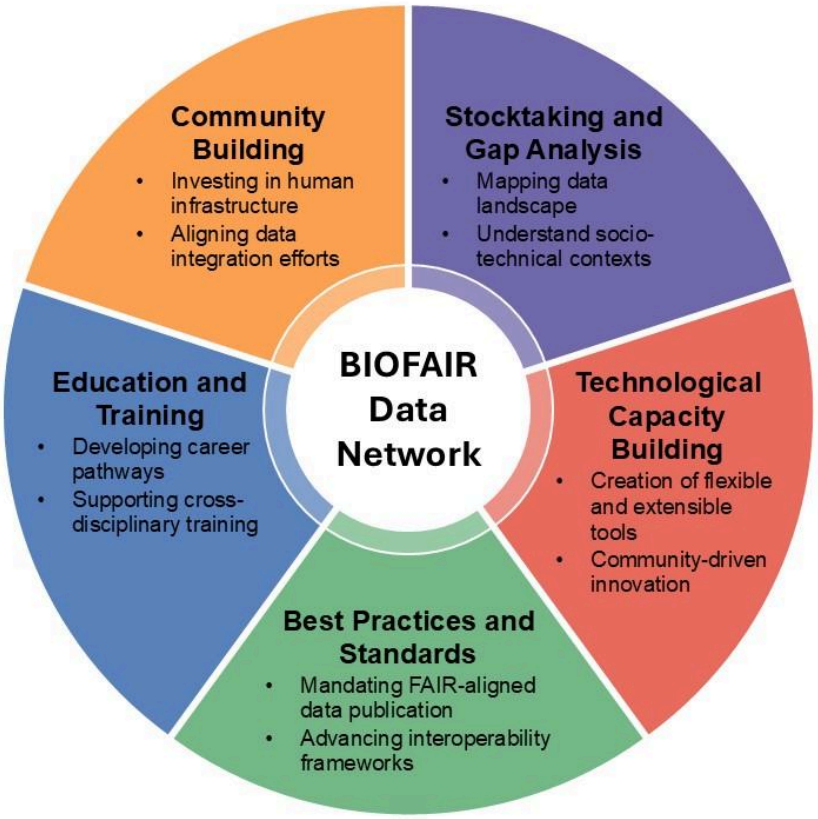


*Figure 4.1. Summative pictograph illustrating the 5 cross-cutting themes of the BIOFAIR Data Network project produced from the Roadmap Exercise held during the interdisciplinary workshop.*

***Stocktaking and gap analysis***
Systematically assess, align, and enhance integration, maintenance, accessibility, equity, training, and best practices across national and global data ecosystems through expert-led working groups with sustained funding by:

- Mapping the data landscape: Identify and quantify all data entities, infrastructures, tools, standards, and vocabularies in use, emphasizing interconnectivity, redundancy, semantics, and discipline-specific variation.
- Analyzing and improving infrastructure sustainability: Evaluate current infrastructure funding models, gaps, and overlaps.
- Assessing reuse and adoption of current tools and standards: Create mechanisms to understand the legacy and effectiveness of existing tools, resources and services, as well as impediments to their widespread adoption, to facilitate the development and alignment of agreed-upon, reusable standards.
- Examining data stewardship practices: Aggregate, analyze, and determine gaps in existing frameworks and best practices for data sharing, access, and protection by coordinating across sectors and including underrepresented voices and local knowledge.
- Evaluating socio-technical contexts: Conduct research to identify and then bridge gaps between intended and actual tool use, ensuring equitable, user-centered design aligns with community practices and expertise.
- Identifying opportunities for capacity building: Survey and address community training needs, skill gaps, and FAIR-related competencies via tailored learning resources developed by diverse expert teams.

***Technological capacity-building***
Strategically invest in integrated, interoperable, and inclusive technological capacity building that enhances data availability, accessibility, maintenance, equity, and usability by:

- Advancing standardization and interoperability: Support the adoption of common metadata templates, data models, and identifier resolution systems. These measures could be enhanced through the use of shared APIs, collaboration with software engineers, and AI or machine learning (ML) tools. They will support data provenance, governance, and integration promoting FAIR principles, and reduce duplication across repositories, content management systems, and domains.
- Investing in collaborative infrastructure development for sustainable and scalable data integration: Collaborate with funding agencies to invest in resilient, FAIR-aligned cyberinfrastructure that supports scalable data integration, reproducibility, and access regardless of location. Promote partnerships—especially inclusive of under-resourced institutions—by funding essential hardware, software, and IT support. Prioritize the sustainability of key, living infrastructure elements such as ontologies, identifier registries, and certification programs, and support the retroactive alignment of existing datasets to common standards to ensure long-term interoperability, equity, and scalability.

- Developing flexible and extensible tooling: Create flexible tools and integration strategies to accommodate emerging technologies and future data types, ensuring the longevity and adaptability of data systems.
- Enabling community-driven innovation: Co-develop infrastructure that incorporates data stewardship standards with journals, repositories, and data scientists. Incentivize repository interconnectivity and shared data stewardship responsibilities through dedicated funding and recognition mechanisms.

***Best practices and standards***

Establish a coordinated, cross-sector effort to define, adopt, and continuously evolve best practices and standards that enable seamless, FAIR-aligned data workflows that integrate across repositories, tools, institutions, geographies, and scientific disciplines by:

- Mandating FAIR-aligned data publication integrated with repositories: Standardize the requirement for authors to publish data concurrently with research outputs via community-recognized portals. Develop shared editorial tools and repository workflows to streamline the integration of data into centralized, FAIR-compliant systems, enabling automatic linkage and citation.
- Optimizing shared standards and interoperability frameworks: Expand and harmonize controlled vocabularies (e.g., DarwinCore), identifier systems (e.g., DOIs, Archival Resource Key or ARKs) (30), metadata schemas, and frameworks for linked data (e.g., DES, Digital Objects Architecture or DOA) (31) to support seamless data exchange.
- Embedding data integration in professional norms and incentives: Develop and disseminate best practices across the research lifecycle—from data collection to publication—by engaging publishers, conferences, and academic institutions. Link adoption of integration standards to professional recognition, certifications, and career advancement pathways.
- Establishing best practices for equity, security, and access in integrated data systems: Develop and provide clear guidelines for data access, protection, and use, including privacy-respecting tiered access models, data visitation, and infrastructure support for regions with limited connectivity. Promote a unified integration approach that includes both openness and responsibility.
- Formalizing iterative processes for the improvement of standards and practices: Create mechanisms for regular community feedback, versioning, and updates to standards and best practices. Encourage the development of tools that track integration quality (e.g., vocabulary alignment, identifier resolution) and facilitate data annotation, attribution, and cross-platform recognition.

***Education and training***

Advance and sustain comprehensive data stewardship, infrastructure, training, and policy integration through coordinated efforts across institutions, disciplines, and funding agencies by:

- Promoting identifier use for accessibility: Advance the adoption of unique identifiers across and beyond disciplines through workshops and a cross-disciplinary advocacy group.

- Strengthening digital asset stewardship: Survey institutional practices and educate the community on digital asset maintenance, emphasizing the importance of biodiversity infrastructures and long-term data care.
- Building human capacity and institutional support: Create and fund roles like data managers and architects, embed FAIR and Open Science into education, and support the development of early training modules (e.g., DarwinCore).
- Supporting inclusive, cross-disciplinary training and engaging community perspectives: Build capacity across sectors through training on standards, metadata, and interoperability tools. Support early education modules, workshops, and a shared knowledge base, ensuring underrepresented communities are actively engaged in shaping integration practices while incorporating cultural and linguistic diversity through partnerships with language experts and AI tools to ensure inclusive data practices and broadened access.
- Developing educational standards, data stewardship certifications, and career pathways: Establish benchmarks, certifications, and a defined data stewardship career framework; pilot accreditation programs in collaboration with key societies and institutions.

***Community building***

Build inclusive, collaborative, and sustainable communities that integrate technical, educational, and policy-driven strategies to support data sharing, access, and reuse by:

- Fostering cross-disciplinary collaboration: Promote engagement and collaboration among data users, providers, and technical experts to promote training in FAIR data principles, data integration and interoperability, and data reuse.
- Aligning data integration efforts: Support research through accessible guidance, distributed technical expertise, and funding.
- Promoting biodiversity data stewardship: Enhance data stewardship through advocacy, targeted outreach, and professional development that increases visibility of careers, FAIR data skills and competencies, and data use success stories.
- Investing in human infrastructure: Develop workforce capacity through long-term support for key roles like data managers and mobilization specialists, providing workforce development, and facilitating community-led knowledge exchange through inclusive stakeholder networks.
- Advocating for policy alignment: Support FAIR data standards in publishing, funding, and infrastructure support, and creation of long-term repositories for the persistence of data, knowledge, and expertise.

## Future directions

In conclusion, the broad recommendations from the BIOFAIR Data Network listening sessions and workshop underscore the urgent need for coordinated, sustained efforts to strengthen the national and global data ecosystems. This includes systematically assessing and improving data infrastructure, stewardship, and policy integration; advancing FAIR data practices through inclusive and collaborative communities; and ensuring equitable access, usability, and sustainability of data. Key strategies involve forming expert-led task groups, investing in

cyberinfrastructure and human capacity, promoting standardization, and aligning efforts across institutions, disciplines, and sectors to foster a more integrated and effective data landscape.

## Chapter 5: Enabling the Future of Data Integration

Biodiversity knowledge in the 21st century is faced with numerous challenges, including the consequences of global change, the lack of stewardship of our natural resources, the growing threat of emerging zoonotic diseases, and an accelerating decline in global biodiversity. Failing to address these challenges poses considerable threats to human health, food security, and other aspects of a postmodern society (32). Significantly advancing our capability to analyze Earth's complex systems and address modern and future challenges will require the integration of biological and environmental data across multiple domains that historically have been disparate from each other, allowing a broad range of researchers to better understand the past, present, and future of life on earth.

### The BIOFAIR Data Network

The BIOFAIR Data Network is envisioned to be an open consortium of biological and environmental data providers, managers, and users that would facilitate the integration of a network of inter-linked data into a technological framework for use in primary research, education, conservation application, and policy. By establishing this data network, we as a community will gain the capacity, flexibility and adaptability to address dynamically arising complex, interdisciplinary research questions that are central to the challenges mentioned above, and more. The BIOFAIR Data Network project is an outgrowth of BCoN's Extended Specimen Network concept, which in turn built on ideas laid out in Webster's ornithological-focused "extended specimen" concept (33). The key concept is that the value of biological specimens increases proportionally to the linkages between those specimens and other types of associated data, as these linkages reveal the factors that allowed that organism to exist in the place and time of its documentation as a specimen. When aggregated taxonomically, geographically and/or temporally, our specimens so extended can lead to new insights into biodiversity and ecosystem function. Equally, the ability to link to tangible examples of life should add an important new dimension to studies that begin as the analysis of environmental data trends.

Workshop participants highlighted a number of research questions (Appendix D) that could be addressed with the development and implementation of the BIOFAIR Data Network. These included: 1) How do species adapt to abiotic and biotic environments?; 2) How do genetics and the environment affect interactions between species and how do genetic x environment interactions influence population dynamics and shape species distributions?; 3) How can we reliably forecast changes in biodiversity?; and 4) How is human health affected by changes in biodiversity and what components of biodiversity are most influential? Participants also suggested that such a data network could be used to, for example, better understand how we can control the spread of invasive species, especially in an increasingly connected and changing world, predict epidemics and pandemics of devastating diseases of crop plants and livestock, and provide more accurate assessments of the environmental consequences of land development and conservation efforts. Such data might form the basis of a digital twin of earth's ecosystems, providing a real-time digital replica of biotic and abiotic factors that would enable virtual experiments to address such questions (34).

The discussions facilitated through the BIOFAIR listening sessions and workshop presentations and roadmapping exercise indicated strong support for increased data integration; at the same time, there was also widespread agreement that there are significant obstacles to realizing this vision (35). The expertise, resources and infrastructures for integrating these diverse data sources and novel interfaces of querying such a broad array of biological and environmental data sources does not exist. The development and stewarding of such infrastructure would most effectively be overseen by individuals committed to working collaboratively to ensure that such infrastructure is comprehensive, accessible and sustainable.

**Other related and on-going projects**
There are of course existing projects working toward the integration of biological and environmental data and thereby addressing, on a project basis, some of the technical and community-building issues that were identified in the BIOFAIR consultations. Several of these projects were presented at, and/or participated in, the listening sessions. Chapter three of this report summarizes the projects that were presented at the workshop. Other notable efforts include the International Partners for the Digital Extended Specimen (IPDES), an ad-hoc group consisting of representatives from organizations including GBIF, iDigBio, AIBS, BCoN, Atlas of Living Australia (ALA), DiSSCo and others that aims to facilitate the coordination of a globally integrated network of stakeholders to build an extensible technical and social infrastructure for the Digital Extended Specimen (DES) vision. IPDES works to address the need for deeper and more frequent communication and collaboration across the globe as biodiversity data providers move towards connecting and linking diverse information. As a network inclusive of various regional, national, and international geo- and biodiversity data-oriented organizations, IPDES is well-poised to contribute to goals and products outlined in the BIOFAIR consultations and roadmap. In addition, the Environmental Bioinformatics group of the Swiss Institute for Bioinformatics (36), which has the goal of developing data science tools and services to contribute to planetary preservation and restoration by combining diverse data sources, such as genomics, species observations, environmental DNA and information from published biodiversity literature. The creation of a technical infrastructure that could link these diverse data sources is the mission of ELIXIR, an intergovernmental organization that brings together life science resources from across Europe. These resources include databases, software tools, training materials, cloud storage and supercomputers (37). ELIXIR also includes a training component, TeSS, which is an online portal that gathers life science training materials and courses from across Europe (38). The liberation of knowledge from the published literature is the primary goal of the Dissentis Roadmap whose vision is that by 2035, "the power of biodiversity knowledge from research publications will be fully leveraged within an open science framework, including unencumbered data discovery, access, and re-use across scientific disciplines and policy applications" (39). Although interoperable networks that include species occurrence data from biological collections seems to be within the scope of these projects, they do not cite collections- and specimen-based networks.

ALA has responded to feedback from users who recommend broadening the scope of interoperable data in their network beyond species occurrence to include changes that will

enable ALA users to model species population trends, as well as absence and abundance, important metrics in successfully managing and assessing the health of ecosystems (40). They are collaborating with GBIF in this work, which has a central task of extending the Darwin Core standard to include a wider range of data types.

In 2022, the Canadian Genomics Research and Development Initiative launched GenARCC. Using genomic tools, GenARCC aims to inform climate change adaptation, to help protect Canada's biodiversity, ecosystem resilience, food security, and health (41). The Environmental Science Innovation and Inclusion Lab (ESIIL), funded by the National Science Foundation, has the goal of enabling a broad range of biological and environmental scientists to work together to address self-selected scientific questions (42). The emphasis in ESIIL is on training in the use of existing tools and creating an environment in which scientists can work across scientific data domains.

These existing and nascent cross-cutting projects represent powerful steps toward addressing the widely acknowledged need to integrate environmental and biological data, and their associated communities to create the new knowledge we need to understand the future of life in a changing environment. However, these projects have not shared their journeys to collaboration in such a way that would provide generalizable guidance for the creation of an overarching BIOFAIR Data Network. Socio-technical studies of other scientific communities, such as that of Vertessi and colleagues to assess the teams that have collaborated to analyse data from space exploration (43) may provide some useful insight, and closer attention to this work may provide some guidance for prioritizing future efforts.

**Collections-based Biodiversity Data**

Since the BIOFAIR consultations were primarily focused on finding commonalities among aspirations and challenges of the data communities we engaged, we did not focus on barriers to the greater inclusion of specimen-based species occurrence data in a broader assortment of environmental data studies. However, we did get some hints about those barriers. It will be a surprise to no one in the collections community that data completeness and consistency are obstacles to the incorporation of specimen data into broader biological and environmental studies; the inconsistency of taxonomic names and the lack of well-documented georeferences are two cited examples. Additionally, users who might want to incorporate specimen data are sometimes not sure about the completeness or quality of the information derived from specimens. For example, if one is interested in incorporating species occurrence data from specimens of insects from Nebraska, one can download the data with little effort, but there is no easy way to know if the digitized data represents all the species that occur in Nebraska, or if the prevalence of one taxonomic group over others represents an actual biological phenomenon or just the digitization priorities as dictated by research interests of digitization grant success. These insights were obtained as incidental comments during the Climate and Environmental Data listening session. Similar discussions focusing on the use of extended biodiversity data in ecological and environmental investigations during the development of the Digital Extended Specimen concept (6) proposed the development of standardized metadata informing data quality (44). To further investigate the barriers to more widespread use of specimen-derived data in analyses and potential solutions, we should consider following the model of the ALA,

which conducted stakeholder focus groups and questionnaires to identify barriers to incorporation of species occurrence data based on specimens into broader environmental data projects and started to implement functionality (e.g., predefined data quality profiles) that improves data usability (40).

To overcome these barriers to the broader use of specimen-derived data, institutions and collections should continue to digitize their collections and to edit and improve the digitized data by providing geocoordinates with appropriate uncertainty radii and updated taxonomic names. Artificial Intelligence tools may help to standardize these data. As recommended in the NASEM Continental Scale Biology report, resources should be allocated for next-generation digitization of biodiversity collections to enhance their utility as reference standards for CSB and to enable the development of digital ecosystem twins (45). This will require new bioinformatics tools that enable access to, and management of, preserved and living collections to facilitate their utility for interpretation of in situ and remotely sensed data.

**Where do we go from here?**

Building a BIOFAIR Data Network is a monumental task that will require collaboration across domains from both within the biological sciences and broadly across other scientific fields central to solving the challenges that face us in the 21st century and beyond. We also recognize that this effort will require long-term investment by the United States government and its agencies to develop and maintain the requisite technological infrastructure and international collaboration to cultivate the social infrastructure to advance this effort on a global scale. The roadmap and vision presented here, developed with cross-domain community input, represents a framework that a community of practice can use to begin filling in the gaps in our current social and technological infrastructure to advance towards the development and implementation of a BIOFAIR Data Network. Up to this point, the United States has lagged behind in its efforts to integrate data across domains compared to other countries. As such, we call on the scientific community, institutions of higher education, and government at both the federal and state levels to take up the torch and carry forward our vision for building an integrated, open, findable, accessible, interoperable, and reusable data network.

## References


(1) Thiers B, Monfils A, Zaspel J, Ellwood, E, Bentley A, Levan K, Bates J, Jennings D, Contreras D, Lagomarsino L, Mabee P, Ford L, Guralnick R, Gropp R, Revelez M, Cobb N, Lendemer J, Seltmann K, & Aime MC. 2019. Extending U.S. Biodiversity Collections to Promote Research and Education. A report by the Biodiversity Collections Network (2019). https://bcon.aibs.org/wp-content/uploads/2019/04/Extended-Specimen-Full-Report.pdf

(2) Lendemer J, Thiers B, Monfils AK, Zaspel J, Ellwood ER, Bentley A, LeVan K, Bates J, Jennings D, Contreras D, Lagomarsino L, Mabee P, Ford LS, Guralnick R, Gropp RE, Revelez M, Cobb N, Seltmann K, & Aime MC. 2020. The Extended Specimen Network: A Strategy to Enhance US Biodiversity Collections, Promote Research and Education. BioScience 70(1):23–30, https://doi.org/10.1093/biosci/biz140

(3) Bentley A, Thiers B, Moser WE, Watkins-Colwell GJ, Zimkus BM, Monfils AK, Franz NM, Bates JM, Boundy-Mills K, Lomas MW, Ellwood ER, Poo S, Contreras DL, Webster MS, Nelson G, & Pandey JL. 2024. Community Action: Planning for Specimen Management in Funding Proposals. 74(7):435–439, https://doi.org/10.1093/biosci/biae032

(4) Nelson G, Elwood L, & Zimkus B. 2024. Envisioning a Natural History Collections Action Center [white paper]. iDigBio. https://www.idigbio.org/content/envisioning-natural-history-collections-action-center

(5) National Academies of Sciences, Engineering, and Medicine. 2020. *Biological Collections: Ensuring Critical Research and Education for the 21st Century*. Washington, DC: The National Academies Press. https://doi.org/10.17226/25592.

(6) Hardisty, AR, Ellwood ER, Nelson G, Zimkus B, et al. 2022. Digital Extended Specimens: Enabling an Extensible Network of Biodiversity Data Records as Integrated Digital Objects on the Internet. BioScience 72:978–987. doi: 10.1093/biosci/biac060

(7) Biodiversity Collections Observation Network. BIOFAIR Data Network. (https://bcon.aibs.org/biofair/). Accessed 1 Jul 2025.

(8) Flood J, Minkler M, Hennessey Lavery S, et al. 2015. The Collective Impact Model and Its Potential for Health Promotion: Overview and Case Study of a Healthy Retail Initiative in San Francisco. Health Education & Behavior 42:654-668. doi:10.1177/1090198115577372

(9) Local Contexts (https://localcontexts.org/#). Accessed 1 Jul 2025.

(10) Ratnasingham S & Hebert PDN. 2007. BOLD: The Barcode of Life Data System (http://www.barcodinglife.org). Mol Ecol Notes. May 1(7):355-364. doi:10.1111/j.1471-8286.2007.01678.x.

(11) Global Biodiversity Information Facility, GBIF (https://www.gbif.org). Accessed 1 Jul 2025.

(12) International Nucleotide Sequence Database Collaboration, INSDC (https://www.insdc.org/). Accessed 1 Jul 2025.

(13) National Ecological Observatory Network, NEON (https://www.neonscience.org/). Accessed 1 Jul 2025.

(14) Long Term Ecological Research Network, LTER (https://lternet.edu/about/). Accessed 1 Jul 2025.

(15) Colella JP, Stephens RB, Campbell ML, Kohli BA, Parsons DJ, & Mclean BS. 2021. The open-specimen movement. BioScience, 71(4), 405-414.

(16) Distributed System of Scientific Collections, DISSCo (https://www.dissco.eu). Accessed 1 Jul 2025.

(17) Davies N, Deck J, Kansa EC, Kansa SW, Kunze J, Meyer C, Orrell T, Ramdeen S, Snyder R, Vieglais D, Walls RL, & Lehnert K. 2021. Internet of Samples (iSamples): Toward an interdisciplinary cyberinfrastructure for material samples, GigaScience 10(5), https://doi.org/10.1093/gigascience/giab028

(18) Wieczorek J, Bloom D, Guralnick R, & Blum S. 2012. Darwin Core: An Evolving Community-Developed Biodiversity Data Standard. PLOS One https://doi.org/10.1371/journal.pone.0029715.

(19) Sica Y, Ingenloff K, Gan YM, & Kachian Z. 2022. Application of Humboldt Extension to Real-world Cases. Biodiversity Information Science and Standards 6: e91502. https://doi.org/10.3897/biss.6.91502

(20) National Academies of Sciences, Engineering, and Medicine. 2024. Foundational Research Gaps and Future Directions for Digital Twins. Washington, DC: The National Academies Press. https://doi.org/10.17226/26894.

(21) Global Genome Biodiversity Network, GGBN (https://www.ggbn.org/ggbn_portal/). Accessed 1 Jul 2025.

(22) Integrated Digitized Biocollections, iDigBio (https://www.idigbio.org/). Accessed 1 Jul 2025.

(23) Ocean Biodiversity Information System, OBIS (https://obis.org/). Accessed 1 Jul 2025.

(24) Vertnet (http://www.vertnet.org/). Accessed 1 Jul 2025.

(25) Biodiversity Literature Repository (https://biolitrepo.org/). Accessed 1 Jul 2025.

(26) Bénichou L, Gérard I, Laureys É, Price M. 2018. Consortium of European Taxonomic Facilities (CETAF) best practices in electronic publishing in taxonomy. European Journal of Taxonomy (475). https://doi.org/10.5852/ejt.2018.475

(27) ArPHA Writing Tool (https://arpha.pensoft.net/). Accessed 1 Jul 2025.

(28) iChatBio (ichatbio.org). Accessed 1 Jul 2025.

(29) Biodiversity Heritage Library (https://www.biodiversitylibrary.org/). Accessed 1 Jul 2025.

(30) Archival Resource Key Alliance (https://arks.org/). Accessed 18 Jul 2025.

(31) Digital Object Architecture (https://www.dona.net/digitalobjectarchitecture). Accessed 18 Jul 2025.

(32) Lopez-Claros A, Dahl AL, & Groff M. 2020. Global Governance and the Emergence of Global Institutions for the 21st Century. Cambridge: Cambridge University Press.

(33) Webster MS, ed. 2017. The Extended Specimen: Emerging Frontiers in Collections-Based Ornithological Research. CRC Press.

(34) Goodchild MF, Connor D, Fotheringham AS, Frazier A, Kedron P, Li W, & Tong D. 2024. Digital twins in urban informatics. Urban Info 3, 16. https://doi.org/10.1007/s44212-024-00048-6

(35) Lave J & Wenger E. 1991. Situated Learning: Legitimate Peripheral Participation. Cambridge: Cambridge University Press.

(36) Swiss Institute for Bioinformatics (https://www.sib.swiss/environmental-bioinformatics). Accessed 1 Jul 2025.

(37) ELIXIR (https://elixir-europe.org/about-us). Accessed 1 Jul 2025.

(38) ELIXIR TeSS (https://tess.elixir-europe.org/). Accessed 1 Jul 2025.

(39) The Disentis Roadmap. 2024. Bouchout Declaration. (https://www.bouchoutdeclaration.org/roadmap-2024/). Accessed 1 Jul 2025.

(40) Belbin L, Wallis E, Hobern D, Zerger A. 2021. The Atlas of Living Australia: History, current state and future directions. Biodiversity Data Journal 9: e65023. https://doi.org/10.3897/BDJ.9.e65023.

(41) GenARCC (https://www.canada.ca/en/environment-climate-change/services/climate-change/adapting/genomic-adaptation-resilience-climate-change.html). Accessed 1 Jul 2025.

(42) Environmental Data Science Innovation & Impact Lab (ESIIL) (https://esiil.org/). Accessed 1 Jul 2025.

(43) Vertesi J. 2015. Seeing Like a Rover: How Robots, Teams, and Images Craft Knowledge of Mars. Chicago: University of Chicago Press

(44) Hansen S, Buschbom J, Karim T, Monfils A. 2021. Building the Digital Extended Specimen: A case study of invasive European frog-bit (Hydrocharis morsus-ranae L.). Biodiversity Information Science and Standards 5: e73814. https://doi.org/10.3897/biss.5.73814

(45) National Academies of Sciences, Engineering, and Medicine. 2025. A Vision for Continental-Scale Biology: Research Across Multiple Scales. Washington, DC: The National Academies Press. https://doi.org/10.17226/27285.

## Authorship of this Document

Andrew Bentley, Anna Monfils, Barbara Thiers, Breda Zimkus, Brooke-Long Fox, Cameron Pittman, David Kunkel, Dori Contreras, Gil Nelson, Greg Watkins-Colwell, John Bates, Julia Portmann, Jyotsna L. Pandey, Libby Ellwood, Matthew Sheik, Michael Webster, Mike Lomas, Nico Franz, Nimanthi Abeyrathna, Sinlan Poo, and William Moser

## Appendices (Separate Document)

**A: List of Participants**

**B: BIOFAIR Data Network Workshop Agenda**

**C: Stakeholders Master List**

**D: What could the BIOFAIR Network enable?**

**E: Refined Roadmap Tables**

**F: Enhanced AI Summary**
