# Supplementary material for: Integrating biological and environmental data to solve key scientific and societal challenges
Source: Bioscience. 2025 Oct 15;76(1):13–20. doi: 10.1093/biosci/biaf150 (PMC12771510; doi:10.1093/biosci/biaf150)
Supplement: biaf150_Supplemental_Files [file biaf150_supplemental_files.zip › BIOFAIR Final Report Appendices.pdf]

Appendix A. List of Participants

Listening Sessions

1. Federal Agencies Listening Session | June 14, 2024

First Name	Last Name	Organization
William	Moser	Smithsonian National Museum of Natural History, Biodiversity Collections Network (BCoN)
Hollie	White	Department of Homeland Security
Diane	DiEuliis	National Defense University
Scott	Miller	Smithsonian Institution
Michaela	Johnson	U.S. Geological Survey (USGS)
Ann	Hitchcock	National Park Service
Andrew	Bentley	University of Kansas Biodiversity Institute, BCoN
Jonathan	Blythe	Bureau of Ocean Energy Management
David	Kunkel	Oklahoma State University, BCoN
Jennifer	Hoguet	National Institute of Standards and Technology
Tera	Hinkley-Bressi	Bureau of Land Management
Rebecca	Gast	U.S. National Science Foundation (NSF)
Brooke	Long-Fox	Phoenix Bioinformatics, BCoN
Julia	Portmann	Map of Life, BCoN
Nico	Franz	University of Kansas, BCoN
Gil	Nelson	iDigBio, BCoN
Barbara	Thiers	Denver Botanic Gardens, BCoN
Allen	Collins	NOAA National Systematics Lab, NOAA Fisheries Office of Science and Technology
Kara	Jones	USGS
Gayle	Volk	U.S. Department of Agriculture (USDA) Agricultural Research Service (ARS)
Taylor	Soniat	Centers for Disease Control and Prevention
Kevin	Hackett	USDA-ARS
Greg	Evans	USDA- Animal and Plant Health Inspection Service
Dave	Vieglais	Smithsonian Institution
Greg	Watkins-Colwell	Yale Peabody Museum, BCoN
Tanja	Grkovic	National Cancer Institute, National Institutes of Health
Reed	Beaman	NSF
Katlyn	Scholl	U.S. Department of State

Sean	Smith	U.S. Customs and Border Protection (CBP)
Anna	Monfils	Central Michigan University, BCoN
Adam	Pitt	CBP
Chris	Meyer	Smithsonian Institution
Matthew	Sheik	Denver Botanic Gardens, BCoN
John	Bates	Field Museum of Natural History, BCoN
Diane	Bosnjak	American Institute of Biological Sciences (AIBS)
Jyotsna	Pandey	AIBS, BCoN, Natural Science Collections Alliance, International Partners for the Digital Extended Specimen (IPDES)

2. **Genetic and Genomic Data Listening Session | June 26, 2024**

First Name	Last Name	Organization
David	Kunkel	Oklahoma State University, BCoN
Michael	Lomas	Bigelow Laboratory for Ocean Sciences /National Center for Marine Algae and Microbiota, BCoN
Andrew	Bentley	University of Kansas Biodiversity Institute, BCoN
Lynette	Strickland	Black in Genetics/Boston University
Taylor	Soniat	CDC
Kevin	Kerr	Centre for Biodiversity Genomics
Nimanthi	Abeyrathna	Clarkson University, BCoN
Cameron	Pittman	Denver Museum of Nature & Science
Joana	Pauperio	EMBL-European Bioinformatics Institute
Débora	S. Raposo	German Federation for Biological Data (GFBio e.V.)/Leibniz Institute DSMZ
Tobias	Frøslev	Global Biodiversity Information Facility (GBIF)
Jose	Lopez	Global Invertebrate Genomics Alliance
Breda	Zimkus	Harvard Museum of Comparative Zoology, BCoN
Jack	Koch	LSU Agricultural Center Aquatic Germplasm and Genetic Resources Center
Sinlan	Poo	Memphis Zoo, BCoN
Conrad	Schoch	National Center for Biotechnology Information (NCBI), National Library of Medicine (NLM), National Institutes of Health (NIH)
Terence	Murphy	NCBI/NLM/NIH
Ryan	Connor	NCBI/NLM/NIH
Eric	Crandall	Pennsylvania State University
Brooke	Long-Fox	Phoenix Bioinformatics, BCoN
John	Bates	The Field Museum, BCoN
Erin	Toffelmier	UCLA/California Conservation Genomics Project
Ann	Mc Cartney	University of California Santa Cruz
Sergey	Nuzhdin	University of Southern California
Tyler	Bourret	USDA-ARS

JC	Buckner	University of Texas Arlington
Diane	Bosnjak	American Institute of Biological Sciences (AIBS)
Jyotsna	Pandey	AIBS, BCoN, Natural Science Collections Alliance, IPDES

3. One Health Listening Session | July 2, 2024

First Name	Last Name	Organization
Barbara	Thiers	Denver Botanic Gardens, BCoN
Bridget	Barker	Northern Arizona University
Chase	LaDue	Oklahoma City Zoo
Cody	Thompson	University of Michigan Museum of Zoology
David	Kunkel	Oklahoma State University, BCoN
Diane	Bosnjak	AIBS
Greg	Watkins-Colwell	Yale Peabody Museum, BCoN
Heather	Skeen	University of Connecticut
Jennifer	Dagostino	Oklahoma City Zoo
Jennifer	Hoguet	National Institute of Standards and Technology
Jocelyn	Colella	University of Kansas Biodiversity Institute
John	Bates	The Field Museum, BCoN
Joseph	Cook	University of New Mexico
Julia	Portmann	Map of Life, BCoN
Jyotsna	Pandey	AIBS, BCoN, Natural Science Collections Alliance, IPDES
Kelly	Speer	University of Michigan
Kendra	Phelps	EcoHealth Alliance
Laura	Goodman	Cornell University
Libby	Ellwood	iDigBio, BCoN, IPDES
Marcia	Revelez	Centers for Disease Control and Prevention
Nicté	Ordóñez	University of Michigan
Paloma	Shimabukuro	GBIF/Fiocruz
Rebecca	Pugh	National Institute of Standards and Technology
Richard Allen	White III	University of North Carolina Charlotte
Sabrina	McNew	University of Arizona
Sinlan	Poo	Memphis Zoo, BCoN
Taylor	Soniat	CDC
Tim	James	University of Michigan
Vincent	Belill	U.S. Customs and Border Protection (CBP)

4. Ecological Data Listening Session | July 12, 2024

First Name	Last Name	Organization
Aaron	David	Archbold Biological Station
Adrienne	Sponberg	Ecological Society of America
Alex	White	Smithsonian Institution
Anna	Monfils	Central Michigan University, BCoN
Barbara	Thiers	Denver Botanic Gardens, BCoN
Brian	Enquist	University of Arizona
Christine	Laney	National Ecological Observatory Network (NEON)
David	Kunkel	Oklahoma State University, BCoN
Diane	Bosnjak	AIBS
Donny	Winston	Polyneme LLC
Dori	Contreras	Perot Museum of Nature and Science, BCoN
Emiley	Eloe-Fadrosh	Lawrence Berkeley National Laboratory/National Microbiome Data Collaborative
Erik	Davenport	National Oceanic and Atmospheric Administration
Erin	Posthumus	USA National Phenology Network
Gregory	Maurer	Environmental Data Initiative, Jornada Basin LTER program
Induja	Mohandas	University of Texas, Austin
Izzy	Hill	U.S. Department of Agriculture
Joana	Soares	Atlantic International Research (AIR) Centre
John	Wieczorek	Rauthiflor LLC
Julia	Kelliher	Los Alamos National Laboratory
Jyotsna	Pandey	AIBS, BCoN, Natural Science Collections Alliance, IPDES
Kate	Ingenloff	Global Biodiversity Information Facility (GBIF)
Kate	Thibault	Battelle, NEON
Leah	Johnson	Los Alamos National Laboratory
Libby	Ellwood	iDigBio, BCoN, IPDES
Maria	Guerreiro	Dryad
Marty	Downs	Long Term Ecological Research (LTER) Network
Matthew	Sheik	Denver Botanic Gardens, BCoN
Mike	Webster	Macaulay Library, Cornell University, BCoN
Moira	Decima	University of California San Diego
Montana	Smith	Pacific Northwest National Lab
Nathan	McTigue	Beaufort Lagoon Ecosystems LTER
Nico	Franz	University of Kansas Biodiversity Institute & Natural History Museum, BCoN
Rachel	Hackett	Michigan Natural Features Inventory
Reed	Beaman	NSF
Shalki	Shrivastava	Lawrence Berkeley National Laboratory
Stephanie	Hampton	Carnegie Science, Ecological Society of America

Stephanie	Parker	NEON, Battelle
Stephen	Formel	U.S. Geological Survey / GBIF-US / Ocean Biodiversity Information System (OBIS)-USA
Susan	Schonberg	The University of Texas Marine Science Institute
Yuri	Corilo	Environmental Molecular Sciences Laboratory, Pacific Northwest National Laboratory

5. **Climate and Environmental Data Listening Session | July 26, 2024**

First Name	Last Name	Organization
David	Kunkel	Oklahoma State University, BCoN
Barbara	Thiers	Denver Botanic Gardens, BCoN
Dori	Contreras	Perot Museum of Nature and Science, BCoN
Anna	Monfils	Central Michigan University, BCoN
Jyotsna	Pandey	AIBS, BCoN, Natural Science Collections Alliance, IPDES
Diane	Bosnjak	AIBS
Ben	Halpern	National Center for Ecological Analysis & Synthesis (NCEAS)
Ty	Tuff	Environmental Data Science Innovation and Inclusion Lab (ESIIL)
Andrew	Bentley	University of Kansas Biodiversity Institute, BCoN
Matthew	Sheik	Denver Botanic Gardens, BCoN
Danielle	Rappaport	University of Maryland, Global Ecosystem Dynamics Investigation (GEDI) Lab
Reed	Beaman	NSF
Corinna	Gries	Environmental Data Initiative
Tongli	Wang	Faculty of Forestry, The University of British Columbia, Canada
Lilly	Jones	Cooperative Institute for Research in Environmental Sciences (CIRES) Earth Lab/University of Colorado Boulder
Patricia	Soranno	Michigan State University
Elise	Zipkin	Michigan State University
Caitlin	Bloomer	University of Illinois Urbana-Champaign
Greg	Husak	Climate Hazards Center, University of California Santa Barbara
Shanan	Peters	University of Wisconsin-Madison
Michael	Belitz	Michigan State University
Jesslyn	Brown	U.S. Geological Survey (USGS)
Rita	Teutonico	Florida International University, Association of Ecosystem Research Centers
Sara	Hansen	Central Michigan University

Erik	Davenport	National Oceanic and Atmospheric Administration
Nichole	Tiernan	Florida International University
Jennifer	Hoguet	National Institute of Standards and Technology
Mira	Anand	HydroSHEDS/Confluvio
Evan	Gallant	Unaffiliated, United Ways of California
Michiko	Beauchamp	USGS - Southwest Climate Adaptation Science Center
Andre	Naranjo	Florida International University
Israel	Borokini	Montana State University Bozeman

6. Biodiversity Informatics Listening Session | August 26, 2024

First Name	Last Name	Organization
Alex	Killion	Yale University - Center for Biodiversity and Global Change
Amanda	Mazza	Duke Lemur Center
Andrea	Thomer	University of Arizona
Andrew	Bentley	Biodiversity Institute, University of Kansas
Arthur	Porto	University of Florida
Beth	Plale	Indiana University
Breda	Zimkus	Harvard Museum of Comparative Zoology
Cameron	Pittman	Denver Museum of Nature & Science
Chandra	Earl	National Ecological Observatory Network (NEON)
Christine	Laney	NEON
Chuck	Cook	Global Biodata Coalition
Claire	Hoffmann	Yale Center for Biodiversity and Global Change
David	Bloom	VertNet
David	Shorthouse	Independent
David	Kunkel	BCoN
Deborah	Paul	Prairie Research Institute, INHS, Species File Group
Diane	Bosnjak	AIBS
Donat	Agosti	Plazi
Dori	Contreras	BCoN, Perot Museum
Edward	Gilbert	Symbiota Support Hub / Arizona State University
Ely	Wallis	Atlas of Living Australia, CSIRO
Erica	Krimmel	N/A
Evgeniy	Meyke	EarthCape
James	Beach	Specify Collections Consortium
James	Macklin	Agriculture and Agri-Food Canada

Jared	Ragland	National Institute of Standards and Technology (NIST)
Jennifer	Hoguet	NIST
Jennifer	Ness	NIST Biorepository
Jessica	Blois	UC Merced
JJ	Dearborn	Biodiversity Heritage Library, Smithsonian Libraries and Archives
Joana	Pauperio	EMBL-EBI
Jörg	Holetschek	Botanic Garden & Botanical Museum Berlin
Jose	Fortes	University of Florida
Juan	Barrios	CONABIO
Julia	Portmann	Yale University
Jutta	Buschbom	Statistical Genetics DE, NHM London UK, International Partners for the Digital Extended Specimen (IPDES)
Jyotsna	Pandey	AIBS, BCoN, Natural Science Collections Alliance, IPDES
Kate	Webbink	Field Museum of Natural History
Katie	Pearson	Symbiota Support Hub / IPDES / iDigBio
Larry	Lannom	Corporation for National Research Initiatives (CNRI)
Libby	Ellwood	iDigBio, BCoN, IPDES
Maria	Praetzellis	California Digital Library
Marie	Grosjean	GBIF Secretariat
Markus	Döring	GBIF / Catalogue of Life (COL)
Michelle	Koo	Museum of Vertebrate Zoology, UC Berkeley, and Arctos Consortium
Mike	Webster	Cornell University
Reed	Beaman	NSF
Rob	Quick	Indiana University
Sam	Leeflang	Naturalis Biodiversity Center
Scott	Miller	Smithsonian
Sharif	Islam	Naturalis Biodiversity Center
Sinlan	Poo	Memphis Zoo
Stephen	Formel	U.S Geological Survey / GBIF-US / OBIS-USA
Steven	Whitfield	Audubon Nature Institute
Sujeevan	Ratnasingham	University of Guelph
Tim	Robertson	GBIF
Torsten	Dikow	Smithsonian National Museum of Natural History
William	Moser	National Museum of Natural History, Smithsonian Institution
Wouter	Addink	Naturalis

Final Workshop

February 13, 2025

First Name	Last Name	Organization
Anand	Narayanan	Florida State University
Andrew	Bentley	Biodiversity Institute, University of Kansas, BCoN
Anna	Monfils	Central Michigan University, BCoN
Barbara	Thiers	Denver Botanic Gardens, BCoN
Breda	Zimkus	Harvard Museum of Comparative Zoology, BCoN
Brooke	Long-Fox	Phoenix Bioinformatics, BCoN
Cameron	Pittman	Denver Museum of Nature & Science, BCoN
Corinna	Gries	Environmental Data Initiative
Daniel	Wildcat	Haskell Indian Nations University
Dave	Vieglais	University of Kansas
David	Bloom	TDWG/VertNet
David	Bloom	TDWG
David	Kunkel	Oklahoma State University, BCoN
David	Shorthouse	Agriculture and Agri-Food Canada
Deborah	Paul	Prairie Research Institute, Illinois Natural History Survey
Diane	Bosnjak	AIBS, BCoN
Diane	DiEuliis	National Defense University
Dianna	Krejsa	Biodiversity Institute, University of Kansas
Donat	Agosti	Plazi
Dori	Contreras	Perot Museum of Nature and Science, BCoN
Ellen	Denny	USA National Phenology Network
Ely	Wallis	Atlas of Living Australia, CSIRO
Eric	Sokol	National Ecological Observatory Network (NEON), Battelle
Gary	Motz	Yale Peabody Museum (Yale University)
Gil	Nelson	iDigBio, BCoN
Greg	Watkins-Colwell	Yale Peabody Museum of Natural History, BCoN
Gregory	Maurer	New Mexico State University
Israel	Borokini	Montana State University Bozeman
Jack	Koch	LSU AgCenter Aquatic Germplasm and Genetic Resources Center
James	Macklin	Agriculture and Agri-Food Canada
Jeannine	Cavender Bares	Harvard University Herbaria
Jennifer	Rudgers	Dept Biology University of New Mexico Castetter Hall
Jianguo (Jack)	Liu	Michigan State University
Joana	Pauperio	EMBL-EBI
Jocelyn	Colella	University of Kansas

John	Bates	The Field Museum of Natural History, BCoN
Jose	Fortes	University of Florida
Joseph	Cook	University of New Mexico
Joseph	Edwards	University of Tennessee - Knoxville
Julia	Portmann	Yale University, BCoN
Jutta	Buschbom	Statistical Genetics, DE, IPDES
Jyotsna	Pandey	AIBS, BCoN, Natural Science Collections Alliance, IPDES
Kate	Ingenloff	GBIF
Katie	Pearson	University of Kansas - Symbiota Support Hub
Katie	Richgels	USGS National Wildlife Health Center
Kendra	Cheruvelil	Michigan State University
Kevin	Kerr	Centre for Biodiversity Genomics
Kim	Miller	USGS National Wildlife Health Center
Kit	Lewers	University of Colorado Boulder
Kyle	Copas	GBIF
Laura	Brenskelle	Integrated Systems Solutions/NOAA IOOS
Libby	Ellwood	iDigBio, BCoN, IPDES
Mariko	Kageyama	Independent
Matthew	Dietrich	Archbold Biological Station
Matthew	Clapham	University of California, Santa Cruz
Matthew	Sheik	Denver Botanic Gardens, BCoN
Michael	Webster	Cornell Lab of Ornithology, BCoN
Mike	Lomas	Bigelow Laboratory for Ocean Sciences, BCoN
Neil	Baertlein	U.S. Geological Survey (USGS) National Wildlife Health Center
Nicky	Nicolson	Royal Botanic Gardens Kew
Nico	Franz	Biodiversity Institute, University of Kansas, BCoN
Paloma	Shimabukuro	Fundação Oswaldo Cruz/Fiocruz
Rachael	Blake	Intertidal Agency
Sara	Hansen	Central Michigan University
Scott	Bates	Purdue University Northwest
Sharif	Islam	Naturalis Biodiversity Center
Shefali	Azad	Archbold Biological Station
Sinlan	Poo	Memphis Zoo, BCoN
Stephanie	Jarmak	NASA Science Explorer (SciX)
Stephen	Formel	USGS
Steven	Whitfield	Audubon Nature Institute
Teferi	Tsegaye	USDA ARS
Teresa	Mayfield-Meyer	Burke Museum
Yasin	Bakış	Tulane University

Appendix B. BIOFAIR Workshop Agenda

Final Workshop: Building the Social and Technological Infrastructure for Data Integration
February 13, 2025 | Zoom Link: https://aibs.zoom.us/j/82134695619
(All times are Eastern Standard)

10:00-11:30 am PART I: Presentations & Discussions on Technological Aspects of BIOFAIR

1. Summary of BIOFAIR Data Network Project
2. Workshop Objectives
3. Invited Presentations
    a. Current data integration efforts at GBIF (Kyle Copas)
    b. Digital Twin and DiSSCo (Sharif Islam)
    c. A Vision for Continental-Scale Biology (Jeannine Cavender Bares)
    d. SciX - Science Explorer Digital Library Portal (Sunny Narayanan)
4. Q&A and Discussion

11:30 am-Noon Break

Noon-1:30 pm PART II: Presentations & Discussions on Social Aspects of BIOFAIR

1. Invited Presentations
    a. Building a community – Collective Impact Model (Kendra Spence Cheruvelil)
    b. Building the MEPA community (Joe Cook)
2. Q&A and Discussion
3. Invited Presentations (continued)
    a. Challenges towards building a global BIOFAIR data network (Israel Borokini)
    b. Indigenous perspective on data sharing (Daniel Wildcat)
4. Q&A

1:30-2:00 pm Break

2:00-4:00 pm PART III: Developing the Roadmap Towards a BIOFAIR Data Network

1. Introduction to the roadmap exercise (15 minutes)
2. Discussion and group work in breakout rooms (85 min)
3. Report out (20 minutes)

4:00-4:15 pm Break

4:15-5:00 pm PART IV: Conclusions and Next Steps

1. Invited Presentation: Looking to the Future
    a. Genomic Adaptation and Resilience to Climate Change - GenARCC (James Macklin)
2. Discussion: Next Steps
3. Conclude

Appendix C. Stakeholders Master List

During the final workshop on February 13, 2025, participants created a master list of key stakeholder organizations and individuals essential to the development of the BIOFAIR Data Network. A Google Spreadsheet version of the list serves as a living document that will continue to evolve and expand over time.

Organization/Agency	Contact Name	Type of Organization
American Institute of Biological Sciences (AIBS)	Jyotsna Pandey	Professional Association/NGO
American Museum of Natural History		Collections Institution
American Society for Microbiologists		Professional Association/NGO
American Society for Virology		Professional Association/NGO
American Veterinary Medical Association		Professional Association/NGO
AmeriFlux		Network
Animal and Plant Health Inspection Service (APHIS)		Federal Agency/Department
Archbold Biological Station		Field Station
Association of Zoos and Aquariums	Shelly Grow	Professional Association/NGO
Audubon Society		Professional Association/NGO
Barcode of Life Datasystems		Database/aggregator
Biodiversity Collections Network	Jyotsna Pandey	Project/Initiative
Biodiversity Information Standards (TDWG)	David Bloom	Standards Development / Network
Botanical Information and Ecology Network (BIEN)		Network
Botanical Research Institute of Texas (BRIT)		Collections Institution
Bureau of Land Management (BLM)		Federal Agency/Department
Bureau of Ocean Energy Management (BOEM)		Federal Agency/Department
Burke Museum		Collections Institution
California Academy of Sciences		Collections Institution
Centers for Disease Control and Prevention (CDC)		Federal Agency/Department
Central Michigan University		Collections Institution
Chan Zuckerberg Initiative (CZI)		Foundation
Coastal and Estuarine Research Federation (CERF)		Professional Association/NGO
Cornell Lab of Ornithology	Mike Webster	Collections Institution
Cornell University Museum of Vertebrates (CUMV)		Collections Institution

Cornell Veterinary Biobank		Collections Institution
CyVerse		Data repository
DataONE		Data repository
Department of Defense		Federal Agency/Department
Department of Energy Biological and Environmental Research		Federal Agency/Department
Department of Energy, Office of Science		Federal Agency/Department
Earth Science Information Partners (ESIP)		Professional Association/NGO
EcoHealth Alliance		Professional Association/NGO
Environmental Data Initiative (EDI)	Paul Hanson	Data repository
Environmental Protection Agency		Federal Agency/Department
Field Museum of Natural History		Collections Institution
Florida Museum of Natural History		Collections Institution
Food and Drug Administration Veterinary Laboratory Investigation and Response Network (FDA Vet-LIRN)		Federal Agency/Department
Forest Global Earth Observatory (Forest-GEO)		Network
Gates Foundation		Foundation
Genbank		Database
Global Biodiversity Information Facility (GBIF)		Aggregator
Global Ocean Observing System Biology and Ecosystem Panel (GOOS BioEco panel)		Project/Initiative
Harvard Museum of Comparative Zoology	Breda Zimkus	Collections Institution
iDigBio		Aggregator
iNaturalist		Professional Association/NGO
International Nucleotide Sequence Database Collaboration (INSDC)		Database
International Partners for the Digital Extended Specimen (IPDES)	Jutta Buschbom	Network
International Society for Biological and Environmental Repositories (ISBER)		Professional Association/NGO
Long-Term Ecological Research Network (LTER)		Network
Marine Biodiversity Observation Network (MBON)	Frank Muller-Karger	Network
Michigan Pathogen Biorepository (M-PABI)		Collection
Moore Foundation		Foundation

MorphoBank	Brooke Long-Fox	Data repository
Museum of Southwestern Biology		Collections Institution
Museums and Emerging Pathogens in the Americas (MEPA)		Network
National Aeronautics and Space Administration (NASA)		Federal Agency/Department
NASA's Distributed Active Archive Centers (DAACs)		Federal Agency/Department
National Bio and Agro-Defense Facility		Federal Agency/Department
National Center for Biotechnology Information (NCBI)		Federal Agency/Department
National Center for Ecological Analysis & Synthesis (NCEAS)		Project/Initiative
National Emerging Infectious Diseases Laboratories (NEIDL)		Research Institution
National Microbiome Data Collaborative (NMDC)		Project/Initiative
National Oceanic and Atmospheric Administration (NOAA)		Federal Agency/Department
National Park Service (NPS)		Federal Agency/Department
Natural Science Collections Alliance (NSCA)	Jyotsna Pandey	Professional Association/NGO
NatureServ		Professional Association/NGO
NIH Aquatic Biomedical Model Repository Network		Network
Ocean Best Practices System (OBPS)		Project/Initiative
Ocean Biodiversity Information System (OBIS)		Aggregator
Ocean Data Information System (ODIS)		Aggregator
Paleobiology Database	Rotating ExCom (alternate: Matthew Clapham)	Database
Partners for Amphibian and Reptile Conservation (PARC)		Project/Initiative
PHAROS - Illuminating the Druggable Genome		Aggregator
PhenoCam		Network
Schmidt Sciences		Foundation
Smithsonian National Museum of National History		Collections Institution
Smithsonian National Zoo and Conservation Biology Institute		Collections Institution
State Department of Environmental Conservation Agencies		Federal Agency/Department
The Consortium of Universities for the Advancemennt of Hydrologic Science, Inc. (CUAHSI)		Professional Association/NGO
The Environmental System Science Data Infrastructure for a Virtual Ecosystem (ESS-DIVE)		Database
The Nature Conservancy		Professional Association/NGO

The Rockefeller Foundation		Foundation
The Society for the Preservation of Natural History Collections (SPNHC)	Greg Watkins-Colwell	Professional Association/NGO
U.S. Culture Collection Network		Project/Initiative
United States Department of Agriculture (USDA) Agricultural Research Service (ARS)		Federal Agency/Department
United States Fish and Wildlife Service (USFWS)		Federal Agency/Department
United States Geological Survey (USGS)		Federal Agency/Department
United States Integrated Ocean Observing System (IOOS)	Gabrielle Canonico	Federal Agency/Department
United States Integrated Ocean Observing System Regional Associations	Jackie Motyka	Federal Agency/Department
University and Jepsen Herbaria		Collections Institution
University of California Museum of Paleontology (UCMP)		Collections Institution
University of California Museum of Vertebrate Zoology (MVZ)		Collections Institution
University of California Natural Reserve System		Field Station
University of Kansas	Andrew Bentley	Collections Institution
University of Michigan Museum of Zoology (UMMZ)		Collections Institution
US National Ecological Observatory Network (NEON)		Network
USA National Phenology Network	Ellen Denny	Network
USDA National Animal Health Laboratory Network (NAHLN)		Federal Agency/Department
Verena		Project/Initiative
VertNet	David Bloom	Aggregator
Wellcome Trust		Foundation
Wildlife Conservation Society		Professional Association/NGO
World Association of Zoos and Aquariums		Professional Association/NGO
World Health Organization (WHO)		International Agency
Yale Peabody Museum (YPM)	Nelson Rios	Collections Institution
Zoo Museum Network (ZooMu)	Gregory Watkins-Colwell; Sinlan Poo; Steven Whitfield; Alexander Shepack	Project/Initiative

Appendix D. What would a BIOFAIR data network enable that is not possible now?

During the workshop, participants were invited to share examples of potential research questions and management applications that could be enabled by the development of a BIOFAIR Data Network. These examples are organized below under the headings *Research Questions* and *Management Applications*, and further grouped by the broad domains they address.

Research Questions

SOME EXAMPLES:

- How do risks associated with invasive species, pathogen emergence, and other shocks such as natural disasters spread across adjacent and distant systems at multiple scales?
- How do ecosystem functions emerge from biological complexity, what mechanisms are involved, and how do these vary across temporal and spatial scales?
- How do human activities and environmental changes (including climate change) in one system generate cascading effects across adjacent and distant systems?

Biodiversity Broadly

- What aspects of biodiversity can we build forecasts for in meaningful ways? What are the relevant forecast horizons that we would have the most confidence in? Are these useful for informing policy? What are the metrics that we should/can forecast? What are the major sources of uncertainty? Can we identify those gaps, and target data collection and integration to address them?
- How does biodiversity in aquatic environments, i.e., moving/fluid environments, change and how does this impact emergent ecosystem functions?
- How many species do we KNOW? What do we know about them?

Evolution

- How do organisms adapt to their abiotic environment (as captured with various kinds of data) and biotic environment (other organisms, and particular coevolution between different species)?
- How are regional patterns of any kind of evolutionary trend in any group of organisms changing through time?

- How does biodiversity (i.e., the number and composition of species in a given area) affect ecosystem function on multiple scales? Are any trends the same as for phylogenetic diversity?
- How does genetic diversity and structure change within taxa/species/lineages across time, specifically for (still) common species and what are the factors/causes?

Organism x Environment Interactions
- How do we leverage long-term ecological datasets to evaluate biodiversity responses to climate change and other stresses across different biomes and ecological regions?
- How do genetics and environment interact to influence pathogenicity?
- How do different interactions among organisms and their environments (e.g., plants and their pollinators and their subsequent/shared physiological tolerances) shape how species are distributed across a landscape and how does this influence population structure at both range edges and within core ranges? How do these factors then connect back to the evolution of these groups?
- What are the historic and ongoing responses to environmental/climate change in a focal group (including deep time)?

Human Focus (Health/Social/Policy)
- What are the human health effects of changes in biodiversity/composition, and what aspects of biodiversity are most influential to human health? (Thinking beyond just zoonotic diseases and into other factors such as air quality, water quality, mental health, or nutritional content of farmed/ranched foods).
- How does the network of data we imagine empower us to better connect with and engage the public as partners? How can we do this in a way to ensure that we are not only talking to ourselves.
- Can we combine social data with environmental data to establish successful and long-term restoration/conservation projects?

Analytical Pipelines
- How can we best leverage multiscale data collected by different disciplines to create instrument-agnostic pipelines? (e.g., how can one see how molecular data connects to spectral or lidar data, how can one explore connections with airborne, ground-based, and space-borne instruments that may have different bandwidths, resolutions, LiDAR pulse rates, etc.)
- How do you link long-term climate and population abundance data (LTER network/NEON) with range limits (and other data from GBIF, iNaturalist, and specimen collections) together with population genomics data to link ecological dynamics with evolutionary consequences?
- How do you link long-term population abundance data (LTER network/NEON) with information on diet (IsoBank, Plant DNA metabarcoding), climate data, etc?
- How do we integrate aspects of traditional knowledge into the data stream?

Management Applications

SOME EXAMPLES:

- Early warning system for emerging pathogens that will issue Doppler radar warnings for locations or periods of time that are high risk for spillovers. How health departments access zoo data to track new zoonotic diseases.
- An environmental data Dashboard that brings together information about biota, biotic interactions, climate, weather, oceans and other waterways, soils, land use, fire, etc.

Monitoring

- Predict which areas will be most at risk (of biological invasions, specific climate change impacts, etc.) in 20, 50, and/or 100+ years.
- With regard to zoos and tracking zoonotic diseases, zoos will have medical records (and likely tissue samples from sample banking efforts). My guess is that this information stays within the veterinary world, and may not get out to biologists. There are cases where zoos do actually track the spread of zoonotics (i.e. West Nile comes to mind). USDA APHIS has a list of reportable wildlife emerging infectious diseases. (https://www.aphis.usda.gov/livestock-poultry-disease/surveillance/reportable-diseases). But, I'm not sure how well this data is linked to other datasets, or whether the data is even accessible beyond APHIS. - See https://zoomunetwork.org/
- Early warning systems can also be developed (and integrated with other data sets) for basic population trends in selected organisms at regional scales.
- Provide the data for the Kunming-Montreal Global Biodiversity Framework monitoring and its Global Knowledge support service for Biodiversity (see eg. https://unep-wcmc.org/en/events/gkssb-webinar-a-global-knowledge-service-for-biodiversity-supporting-parties-needs-for-monitoring-implementation-of-the-gbf).
- Early warning systems - who do they serve? e.g., waste/run-off water surveillance or earthquake warning systems used by those in Japan.
    - Who do they serve? Depends on what the early warning system is for:
        - Can be the public or certain sectors
        - Ex: harmful algal bloom early warning systems can benefit the public or industries like aquaculture; https://ioosassociation.org/nhabon/
        - During the earlier days of the pandemic, there were apps that would track your movements and tell you if you encountered any infection hotspots during the day. This sort of tech could be repurposed to other uses.
- It would be interesting if AI could make use of all those doorbell cameras to track wildlife activity.
- Enable rapid response systems: Today, data, repositories, tools, and work environments are piecemeal - a translation engine/work environment that will allow flexible assembly of large, multidimensional data sets seems needed.

Centralized Technological Infrastructure

- The Biological Collections Action Center highlighted in the NASEM report and identified as a priority in the CHIPS and Science Act of 2022 could function as a centralized HUB for managing these technological and social efforts
- Can we build these networks as local-to-global so that we can continue our work even if different administrations step into place? What makes the research network robust and manages this risk?
- Identify a framework where funding by one agency for the management of one ecosystem level (e.g., fisheries) is formally connected to funding from a different agency that funds the foundation (e.g., phytoplankton) for the managed ecosystem level.

Social Infrastructure
- Development of interdisciplinary and collaborative teams/networks
- Policy leverage
- Develop best practices in drafting data management plans, data use agreements, etc.
- Ecologists and geoscientists in many networks (LTER, CZO, etc) are frequently in the field collecting things, but are not always aware of or well-trained in museum/collections science, and not always well connected to museums or natural history collections themselves. There needs to be an effort to train these collectors to make their samples/specimens more useful (collection techniques, appropriate metadata) and connect them to facilities that would be willing and able to preserve & share what they collect.
- How do we share knowledge across disciplines in a way where they do not need to know the discipline fully to be able to understand what the data is and how to appropriately use it?
- Outreach events and programs (e.g. community, high school, college, etc.)
- Develop sustainable funding mechanisms to reduce reliance on one or two agencies/mechanisms and distribute risk across the network

Software Development
- Developing computational models that can integrate different metadata and simulate/extrapolate different scenarios. (for e.g. climate change impacts, ecosystem adaptations, etc.)
- A platform for globally unique IDs for specimens and associated samples
- Can we provide a bibliography of life (like a specimen's CV) as a starting point for AI applications (e.g. LLMs) including all the publications in a machine-actionable format and some of it prepared for training the system to ask questions such as which virus is hosted by which animal or plant? Where has which specimen been used? Where did specimens collected in a geographic area end up in which collection?

Collections Specific
- Identify how specific specimens have been referenced/used for research (i.e., measuring the impact of specimens and collections).
- Interactive analysis and overlay of data and metadata for samples (towards holistic collections)

Appendix E. Refined Roadmap Tables

Overview

The following tables present the outcomes of the roadmapping exercise conducted during the BIOFAIR Data Network workshop to develop a community-informed plan for building a FAIR, open, and integrated biological and environmental data network. Workshop participants were divided into six breakout groups (Groups 1–6), each focused on one of six priority needs identified during the earlier listening sessions. Each group was tasked with completing a table outlining strategies, short-term outcomes, and long-term outcomes for three assigned milestones.

After the workshop, the raw tables were cleaned, organized, and refined for clarity and consistency by a subgroup of the steering committee. This subgroup also conducted a cross-group analysis, identifying several recurring themes that emerged across all six groups. These common themes include stocktaking and gap analysis, technological capacity building, best practices and standards, education and training, and community building. Each strategy proposed by participants is tagged below with the theme(s) most closely aligned with it.

Group 1 - Enhance the availability of biological and environmental data to support research and decision-Making

**Milestone toward *Increased Data Availability*:**	**Strategies to reach Milestone through measure by resources (a)**	**Short-Term Outcomes (< 5 years) (c)**	**Long-Term Outcomes (+5 years) (d)**
**Expand availability of data types including acoustic data, observational data, and small, focused datasets.** (1)	Create a master catalog of data types through creation of an open-access online searchable catalog of data types by a dedicated group who will define the structure of the catalog and reach out to the various data communities. [Stocktaking and Gap Analysis]	Partially pre-curated catalog made available for input.	Catalog with 80% or more of data types with reference to their structure and implementations.
	Change how data management plans inform a 'what should be available' data catalog through generation of an online form that identifies what should be available based on what was funded by supporting a dedicated data management office. [Stocktaking and Gap Analysis]	Identification of a 'champion' to write an appropriate proposal to the appropriate funding agency for consideration.	Development and release of a single source website that efficiently manages data from funded projects.

	Require authors to concurrently publish data to appropriate FAIR portals/systems with their work in a journal through increasing number of publications devoted to particular data types/sources (e.g., taxonomy, phylogeny, inventories) and/or those journals that publish semantically enhanced data types by developing editorial resources for direct data upload to an appropriate portal, e.g., GBIF, and a repository for the compiled resources (e.g., Biodiversity PubMed Central and SciX)<br>[Best Practices and Standards]	X number of journals with hosted portals (e.g. European Journal of Taxonomy at GBIF) for publications and data (journals and other data repositories).	Make it a standard approach for publications to directly upload the data from their work.<br><br>Convince enough journals to adopt this approach to make it more widely accepted.
	Collate and incorporate information from environmental risk/conservation surveys conducted by land management and other managing authorities through increasing the number of published datasets from these surveys and/or number of agency/survey programs publishing data by creating a common databasing structure that allows for automatic connections across organizations, robust training for how to collect and then utilize data, and prioritizing that the data be discoverable and user-friendly.<br>[Best Practices and Standards]	Produce a protocol, or system, for gathering participation and sharing data at a regional scale from partners in these respective regions.	Growing participation by land management agencies and conservation organizations. Enough data coming into the databasing structure to start to develop helpful analyses, pipelines, and inferences for these data contributors.
**Improve the established culture of collaborative and open data-sharing.**<br>*(2)*	Development, with participation of journals, of more robust and widespread accreditation and attribution mechanisms to acknowledge data provenance and use through standardized data collection and processing metadata templates and issuance of DOIs by developing artificial Intelligence/machine Learning protocols to facilitate application of FAIR principles.<br>[Technological Capacity-Building]	Increase the fraction of datasets with DOIs that are citable and attributable.	Reach 100% of published datasets having associated DOIs.
	Create FAIR data that can be cited and metrics to measure usage provided through generation of a minimum standard format for data sets and metadata that includes a common vocabulary by supporting people to evaluate data centers in other scientific fields for examples of how standardization and common vocabulary is being done and develop a whitepaper for targeted funding.<br>[Best Practices and Standards]	Create a standard data model and identify tools to create standard data outputs to be stored. Create Artificial Intelligence/Machine Learning based toolboxes to include quality control mechanisms within the standard data format.	Create and advocate for a standard format for data sharing (e.g., Darwin Core).
	Create a culture that continually reinforces collaboration, especially when using open data	Create a clearinghouse for tracking the FAIRness of open datasets and their use.	Establish common practice for including these collaborations more openly in presentations and

	sets, through tracking of conference contributions that demonstrate the impact of FAIR data on furthering science and creating educational/training resources that reinforce future generations' FAIR data policies by supporting mechanisms for data users to collaborate with the original FAIR data provider who has a better in-depth understanding of the original data.<br>[Community Building]		publications.<br>Create training infrastructure for students, such as open-access online courses.
	Enable data repositories to proactively support FAIR standards (e.g., establishment and sharing of metadata) through tracking the frequency of datasets that are aligned with FAIR standards at each data repository by committing human and financial resources to support data repositories' efforts to align their own structures with other data repositories, thus making them interoperable.<br>[Best Practices and Standards]	Increase the fraction of datasets at data repositories that are FAIR standard compliant.	Create a system where repositories have a common format to facilitate the mirroring of the same dataset across multiple repositories (e.g., LTER data going to multiple repositories).
**Enhance data integration with existing, compatible data infrastructure projects.**<br>(3)	Incentivize repositories to create connections between adjacent data repositories through formal connections and interoperable systems between repositories by fostering a shared interest in data integration.<br>[Technological Capacity-Building]	Dedicated repositories used by scientists.	Repositories cross-linked and interoperable.
	Develop a common data model between repositories through clearly articulated and publicly available cross-repository data sharing models by supporting dedicated data managers for the development and integration of this common data model.<br>[Technological Capacity-Building]	Common data model developed.	Common data model implemented.
	Discuss with funding agencies the appropriate investment in both data storage, integration and data management (for the agency funded projects). Through requests for proposals from agencies soliciting data management and structures by deployment of resources related to areas requiring high priority attention<br>[Education and Training]	X amount of investment	2X amount of investment
	Make research questions the major motivator for prioritization of data integration efforts through identification of priority research areas by generating accessible publications within the	Identification of "3" research topics extending from what is currently focused on.	Have these "3" primary research topics integrated into the common data infrastructure.

	related domain that recommends funding for the development of links between infrastructure projects.<br>[Community Building]		

Group 2 - Improve capacity for data integration

**Milestone toward *Increased Data Integration*:**	**Strategies to reach Milestone through measure by resources (a)**	**Short-Term Outcomes (< 5 years) (c)**	**Long-Term Outcomes (+5 years) (d)**
**Encourage the use of resolvable persistent identifiers throughout the data lifecycle and document detailed metadata**. [from individual specimen DOI's to datasets and data that have been aggregated]<br>(1)	Select a single, resolvable, community-based identifier through uptake in use by providers, data aggregators, publishers, and other stakeholders through providing funding to utilize a centralized data infrastructure for minting and establishing partnerships for social and technical advocacy.<br>[Best Practices and Standards]	Pool known resolvable, minted identifiers currently in use to begin assessing duplicate assigning of identifiers, as well as data types with no current identifiers to begin advocacy of the use of single identifiers.	Utilize the knowledge gained to begin coordinating the centralization of single-use identifiers (bottom-up and top-down approaches).
	Highlight the importance and need of unique identifiers across disciplines and coordinate the use of identifiers beyond our discipline through conducting discipline-specific workshops focused on the importance and use of unique identifiers by establishing a cross-disciplinary working group of scientists interested in this kind of advocacy.<br>[Education and Training]	Establish a working group with representatives from many disciplines to focus on bettering our understanding of people identifiers and creating a training document on how to create a unique person identifier as a standard operating procedure.	Leading several discipline-specific workshop trainings on establishing mechanisms/strategies for the creation and use of unique identifiers.
	Seek advice from software engineers working with distributed systems on how they manage the challenge of persistently identifying and reassembling disparate data and apply it to our siloed systems through keeping track of the number of databases able to be coupled by establishing relationships with software engineers and providing training for discipline-specific scientists for this kind of work.<br>[Community Building]<br>[Technological Capacity-Building]	Establish quarterly meetings with software engineers to begin establishing standards or methodologies and begin coupling databases.	Network of software engineers and those persistently applying/using unique identifiers help advocate and adopt standards of applying methodologies across discipline-specific, disparate data. These standards would act as training materials.

	Identify all entities/elements that need identifiers, including individual records, datasets, agents, etc., through compiling the numbers of entities by the creation of methodologies for coalescing this data.<br>[Stocktaking and Gap Analysis]	The creation of a catalogue for all elements/entities' unique identifiers is used for and a description of both (which may have been done through existing initiatives already)	A complete catalogue for elements/entities and their unique identifiers that is easily shared across disciplines.
	Mitigate multiple organizations minting the same entity through cataloging identities with multiple unique identifiers by enhancing communication across data aggregators and users, and providing funding to develop tools that can identify these kinds of issues.<br>[Community Building]	Establish a standard format for how unique identifiers should appear in individual databases and data aggregators.	Targeted consolidation of many datasets with multiple unique identifiers applied to them.
	Create a resolving service(s) for existing identifiers through counting the number of existing identifiers by establishing collaboration with computer scientists to create and host a unique identifier resolving service.<br>[Technological Capacity-Building]	Determine if a resolving service must be developed or if there is existing infrastructure with this capability.	Get a resolving service functional and provide support across disciplines for targeted individuals identified as in need of resolution.
**Enhance and advocate for common ontologies that have been adopted by the global scientific community.**<br>(2)	Enhance the DarwinCore vocabulary and its extensions to broaden fields for more interoperability with other standards developed through taking a retroactive approach to identify what terms are being used in data repositories like GBIF and work backwards to map terms with existing DarwinCore vocabulary and extend DarwinCore where needed by advocating for Darwin Core and providing funding to help repositories implement Darwin Core if not currently used.<br>[Best Practices and Standards]	Connect DarwinCore developers with domain-specific scientists to discuss current and future plans to use/extend/expand controlled vocabularies.<br><br>Identify other standards developed by other disciplines.<br><br>Link DarwinCore developers with the developers of other standards to begin conversations on interoperability.	Expand accepted controlled vocabularies for mandatory elements, for DarwinCore.<br><br>Create a workflow that can automate the mapping of controlled vocabularies across discipline-specific standards and DarwinCore.
	Build more bridges across constituents that contribute to biodiversity informatics through conducting a system of user analysis to quantify infrastructure currently being used by establishing a philosophical mind shift to work across siloed data.<br>[Community Building]	Establish a better social network for cross-group coordination/communication	Establish a working group to begin coalescing similar vocabularies across biodiversity informatics data.
	Enhance controlled vocabulary methodologies to include controlled vocabulary metrics in data cleanup metrics already developed through the adoption of methods over time by the development of translation tools to map uncontrolled terms to a controlled vocabulary.<br>[Best Practices and Standards]	Create a registry of controlled vocabulary. Informed by looking at data published to GBIF and extracting values used (column headings).	Create tools that translate/map uncontrolled terms to controlled vocabulary (potentially an AI tool?)

**Conduct a gap analysis to pinpoint key sources, uses, and deficiencies in biodiversity data.** (3)	Conduct a socio-technical ethnography of communities through a grounded theory thematic analysis and a telemetric/log analysis of how tools are being used versus how they were intended and where/if to meet in the middle between UI and UX by identifying expertise in socio-technical ethnography studies. [Stocktaking and Gap Analysis]	Bring together professionals to conduct socio-technical ethnography and begin analysis of findings.<br><br>What data is being leaned on and accessed the most across disciplines (i.e., what is remote sensing, ecology, and natural history leaning on?). What data is being left out? Where are the gaps in the science in terms of data being fed to models, to aggregators, etc?	Using new understanding of what data types are not FAIR, to begin advocating/communicating with those domain-specific scientists to begin establishing FAIR principles and incorporating data into broader repositories.
	Conduct a semantics gap analysis through quantifying the use of semantics and identifying the number of discipline-specific vocabularies and vocabularies used across disciplines by bringing in and working with linguistic/semantic folks. [Stocktaking and Gap Analysis]	Conduct semantic gap analysis and distill findings into a cohesive and shareable format.	Begin filling in gaps across vocabularies (exploring the use of AI in this task) to enhance discoverability and linkages of data types.

Group 3 - Establish sustainable funding models and streamlined infrastructure to ensure the long-term preservation, accessibility, and usability of key biological and environmental data resources

**Milestone toward *Maintenance of Existing Data Resources*:**	**Strategies to reach Milestone through measure by resources (a)**	**Short-Term Outcomes (< 5 years) (c)**	**Long-Term Outcomes (+5 years) (d)**
**Establish sustained support for the maintenance and enhancement of data resources and infrastructure.** (1)	Establish agreement on a single identifier system through adoption by all CMSs, aggregators, publishers, and data users by providing funding for DOIs, DES infrastructure for linking, TDWG for standards development and bioinformaticians [Best Practices and Standards]	Coalescence around a single identifier system	Adoption of identifier by all
	Identify redundancies and gaps in existing infrastructure resources & aggregators through the creation of a diagram of players, their key strengths, and similarities and differences among them, by forming a task group that organizers workshops with major cyberinfrastructure leaders and had funding for upkeep [Stocktaking and Gap Analysis]	Produce the diagram<br>Begin conversations about where some efforts can be consolidated or partitioned (if any)	Consolidate or niche partition some infrastructure efforts and funding mechanisms

	Educate institutions on the need for continued maintenance of ALL digital assets through surveying and documenting current institutional practices and educating the community about best practices based on survey findings<br>[Education and Training]	Data-holding institutions understand the need for continued funding to sustain digital infrastructures.	Institutions follow best practices to sustain their chosen digital infrastructures.
	Make cyberinfrastructure resilient to technical, economic, and other failures through providing mechanisms for data replication, synchronization, relocation, access independent of hosting location by a collaboratively funded project and small institutional awards for widespread adoption<br>[Technological Capacity-Building]	Prototype involving a few institutions and one or more aggregators.	Wide adoption by many institutions, including many under-resourced ones.
	Identify viable distributed funding models for widely used infrastructures and get help from experts (i.e., businesspeople) to implement them by providing incentivized partnerships with business professionals<br>[Stocktaking and Gap Analysis]	Viable funding models and best practices for biodiversity infrastructures identified	Infrastructures start using funding models, with consultation from business professionals
	Communicate the importance of biodiversity infrastructures with a broader audience, including policymakers, through surveys that assess familiarity of key infrastructures among scientists, educators, and other sectors, by communication training, connections with policy makers, and documentation of value, e.g. https://www.gbif.org/value<br>[Education and Training]<br>[Community Building]	List of places and communities that have been surveyed and places where information can be found	Better inclusion of biodiversity in policies, reach people who could provide sustainable funding sources
	Identify data resources and infrastructures, their funding models, and assess the sustainability needs of each through the creation of a living spreadsheet by the formation of a funded task group<br>[Stocktaking and Gap Analysis]	Spreadsheet of data resources and infrastructures, including funding source and sustainability needs	Ongoing maintenance of spreadsheet and online place for it to be accessed
	Invest in human capital for cyberinfrastructure maintenance and development, to include digital infrastructure as well as institutions housing and managing collections, through the creation or enhancement of full-time data managers, data mobilization specialists, and data architects, by recognizing the need for such positions and incorporating them into hiring plans, providing training programs, and establishing sustained funding lines	Improvement of existing cyberinfrastructure resources (or implementation)	Robust cyberinfrastructure, interoperable, integratable

	[Community Building]<br>[Education and Training]		
**Nurture a culture of FAIR data and open science that facilitates better linking of specimens and data in publications.**<br>(2)	Incorporate FAIR and OS into education and training, from undergrad through to established professionals, and reinforce supporting tools, policies and efforts, through the creation of standardized coursework that includes best practices for publishing, ORCiD, general technical operations etc., by creating a working group to develop and maintain up-to-date educational resources<br>[Education and Training]	Established resources for educators to use, people in science have and use an ORCiD and properly cite specimens and data.<br><br>Established requirements for advancement and promotion that incentivize critical solutions that advance FAIR/open data practices<br>● ORCiD<br>● Specimen citation (doi)<br>● Linked open data	Widespread familiarity with FAIR principles among early career workers and students, which will propagate upwards<br><br>People doing science get attribution for their work and collections get attribution for their contributions to science.<br><br>More institutions participating in data sharing (=more data and gaps filled); specimens being accessible, being used more broadly, and being referenced<br><br>Disincentivization of exclusive, paywalled publications. Design for open science.
	Require publishers, professional associations, and conferences to be FAIR and Open, and remove barriers for researchers to adhere as well, through the creation of broadly disseminated best practices and standards for the full research workflow, including citing and publishing datasets, by getting buy-in from publishers and developing the cyberinfrastructure tools and framework to harvest linked data<br>[Best Practices and Standards]	Adoption by at least 10 top professional societies and their flagship publications/conferences<br>Data providers are notified when their data are used in a publication and get enhancements to their data as a result of research using it.	Widely accepted/practiced publication policy.
	Take advantage of GBIF hosted portals to promote and ease implementation of best practices through publicizing them and encouraging their use<br>[Best Practices and Standards]	At least 10 new hosted portals	Accessible data becomes the norm
	Provide cyberinfrastructure support for under-resourced institutions to help them overcome initial barriers and demonstrate an increasing number of online specimens by funding support for hardware and software needed for digitization as well as IT experts who can help chart a path forward<br>[Technological Capacity-Building]	Develop a “pitch” or justification for collections staff to use to gain institutional support for moving towards open science practices.<br><br>Facilitate easier pathways for under-resourced institutions to participate	Increase in number of under-resourced institutions with online data
**Identify and establish common practice that streamlines infrastructure across existing aggregators.**<br>(3)	Darwin Core and DwC Archive education for undergraduates or earlier, by funding for TDWG to develop educational modules<br>[Education and Training]<br>[Best Practices and Standards]	Everyone in the community is familiar with our data sharing model - Darwin Core and how it is used by aggregators - Darwin Core Archive	The biodiversity community has a shared data model to increase interoperability and openness

	Develop ontologies that facilitate data sharing between providers and aggregators, along with the data interoperability that would make this possible, APIs for downstream use, through training people and providing ongoing support via a central organization<br>[Best Practices and Standards]	List of existing ontologies<br><br>A small number of ontologies are developed and their use is adopted	Ontologies are updated as needed<br>Training is provided to new ontology users
	Initiate a task group with members of aggregators, to map out existing methods and best practices and document steps to merge infrastructures to a common standard, by working with TDWG<br>[Stocktaking and Gap Analysis]<br>[Best Practices and Standards]	Data aggregator community to work towards common standards	Data aggregators with shared infrastructure allowing for easy interoperability
	Ensure human- and machine-operable languages and frameworks permit extensible annotation and reuse of data through partnerships among vested parties<br>[Technological Capacity-Building]	Development of data continuity plans and governance models that involve all relevant partners	Ongoing support for partners contributing to the framework

Group 4 - Build a robust training infrastructure to equip researchers, data managers, and early career scientists with the skills needed for the effective use, sharing, and maintenance of biological and environmental datasets

**Milestone toward *Building a Robust Training Infrastructure*:**	**Strategies to reach Milestone through measure by resources (a)**	**Short-Term Outcomes (< 5 years) (c)**	**Long-Term Outcomes (+5 years) (d)**
**Develop and integrate educational curricula and training resources.**<br>(1)	Survey the community to identify key data management skills and competencies, summarizing results, response numbers, and insights. A team of experts will design, distribute the survey, and offer incentives and networking opportunities to boost participation.<br>[Stocktaking and Gap Analysis]	White Paper associated with the results of the survey.	Implementation plan/Best Practices surrounding data management.
	Identify communities, their needs, and learning objectives based on roles and perspectives. Summarize survey results, response numbers, and insights. A working group will survey scientific	Create a directory for various communities, outlining their roles and specific needs. Additionally, form a network of mentors with expertise in data management to support ongoing learning and	Publish a report compiling survey results and detailing the needs of various communities. Additionally, create a learning map that encompasses resource needs and goals, providing

	communities, create an online directory, and publish findings.<br>[Stocktaking and Gap Analysis]	development.	a clear guide for development and training.
	Identify existing resources, gaps, and needs related to FAIR Data principles, compiling a list of available resources and gaps. A working group with software expertise will develop a website or online resource to address these needs.<br>[Stocktaking and Gap Analysis]	Create a centralized location to list and regularly update these resources, including open educational resources like QUBESHub and Data Carpentry, ensuring easy access for the community.	Develop an online system with a form that recommends resources tailored to an institution's goals. Additionally, create accessible pathways to data that minimize the need for data curators, ensuring ease of use and broad access.
	Develop benchmarks and certifications with standardized expectations and educational criteria. Identify X key societies to involve, and form a team with government policy expertise to explore pathways for creating an accreditation program.<br>[Education and Training]	Meeting between stakeholders from different communities to develop a potential accreditation system	Creation of an accreditation system with initial member institutions
**Define and hone career pathway for data stewards.**<br>(2)	Develop a Data Stewardship career pathway framework, defining translatable skills, role descriptions, competencies, and educational requirements. Create a collaborative platform with stakeholders, including data managers and experts, to document the framework.<br>[Education and Training]	A document or standard that exhibits the pathway for data stewardship.	A new generation of Data Stewards enter the field with a full understanding of what their career encompasses.
	Recruit mentors from the data stewardship community, ensuring diversity across sectors, to support and educate early career professionals and students, building a pool of dedicated mentors for ongoing development.<br>[Community Building]	Establish a mentorship network with at least 10 mentors and 30 mentees.	A new generation of Data Stewards enter the field with a large support network of fellow, more-established data stewards.
	Collaborate with the educational community on current initiatives, organizing quarterly webinars, workshops, or networking events for data stewards to share experiences and discuss best practices, leveraging professional networks and partnerships for support.<br>Community Building]	Create a model for best practices surrounding data stewardship and the relationship between research and academic institutions.	Providing opportunities for data stewards to network and share experiences will foster a stronger community and improve the overall effectiveness of data sharing and management.
	Develop interdisciplinary training pathways by partnering with educational institutions or platforms (e.g., Coursera, edX) to create content and integrate it with learning management systems. Form a working group with expertise in education, software, and data stewardship to align content with industry needs and standards.<br>[Technological Capacity-Building]	Develop and launch at least 5 core training modules, each focused on a critical aspect of data stewardship (e.g., data governance, data sharing, FAIR principles).	Creation of an online or in-person curriculum that covers essential skills for data stewards, including data management, metadata standards, data sharing protocols, and ethical considerations for biological and environmental datasets.

**Identify mechanisms for incentivizing training in data science.** (3)	Develop a data science accreditation and certification system, tracking at least 100 participants in the first year. Launch an incentive program within six months and monitor engagement for 12 months. Trial a state-wide accreditation effort, collaborating with funding bodies, academic institutions, and industry partners to secure resources and sponsorship. [Education and Training]	Create a structured incentive program to encourage researchers and early career scientists to participate in data science training. This could include funding for training, certification rewards, and recognition through career advancement opportunities.	Establish at least three distinct types of incentives (e.g., monetary support, certification, career advancement recognition) and
	Create career success models and pathways, aligning educational products with professional outcomes. Collaborate with institutions and employers to make data science skills a requirement or strong incentive for promotion and tenure in research roles. Form a diverse working group to develop and refine these career pathway models. [Best Practices and Standards]	Develop career progression models that explicitly include data science training and establish data science as a required skill for at least 5 key job titles in the field within the first year.	Collaborate with HR departments, research institutions, and universities to integrate data science training into their existing career development frameworks.
	Promote transparency in the job field and highlight the transferable skills of data science across disciplines. Reach 500 individuals through an outreach campaign (e.g., webinars, social media, newsletters), tracking training program enrollments. Form a team of Data Champions to lead the campaign and showcase data stewardship as a valuable skill. [Community Building]	Launch a targeted outreach campaign to highlight the importance of data science training for biological and environmental research, showcasing the benefits and career advancement opportunities.	Raising awareness of data science training opportunities will increase participation and commitment to learning, fostering a culture of continuous skill development.

Group 5 - Develop mechanisms for addressing inequities in data access and policy to ensure the ethical use of data

**Milestone toward *Data Equity and Ethical Use*:**	**Strategies to reach Milestone through measure by resources (a)**	**Short-Term Outcomes (< 5 years) (c)**	**Long-Term Outcomes (+5 years) (d)**
**Identify and develop guidance for equitable and accessible data and technology for all stakeholders.** (1)	Identify and aggregate existing best practices and resources on data management and their originating organizations with the help of a working group composed of representatives from all data domains. [Stocktaking and Gap Analysis]	Areas of overlaps and gaps among communities identified	Comprehensive and publicly available list of best practices

	By leveraging language experts, translational tools, AI, and adaptable templates, develop an enhanced understanding of diverse cultural and linguistic considerations, while recognizing regional and local communities, ensuring appropriate representation, and acknowledging various naming conventions. [Technological Capacity-Building]	Establish a tool for language translation (e.g., GBIF); success in use of guidance	Localization of infrastructures and vocabularies; increasing use of guidance
	Develop guidance on hardware, software architecture, and internet bandwidth to enhance accessibility and increase user engagement, supported by a dedicated working group with expertise in these technical domains. [Best Practices and Standards]	Successful use of guidance by local and/or grassroots organizations	Best practice use is norm for all stakeholder groups
	Identify a diverse stakeholder community that continues to grow and evolve over time with the help of a working group that has representation from different domains. [Community Building]	Preliminary list of stakeholders	Expanded list of stakeholders that evolves over time and allows communities to easily connect to resources
	With the help of educators and data equity experts, develop training and curricula on data use that incorporate existing guidance, and implement capacity-building initiatives, such as train-the-trainer programs, to support the effective uptake and application of this guidance. [Education and Training]	An initial group of trained individuals who can train future cohorts	Curricula is used by all stakeholder groups
**Establish international standards and recommendations to facilitate data access and sharing.** (2)	Identify and compile a list of existing frameworks and standards for data access and sharing that quantify the number of fields/dimensions/sectors covered with the help of an umbrella organization that facilitates regular coordination and evolution of this resource. [Stocktaking and Gap Analysis]	Online resource/library that makes existing frameworks and standards discoverable	Evolution of resource to increase functionality, add analytical features, and improve depth of understanding; established procedures and documentation
	Gap analysis of existing frameworks and standards to identify commonalities, differences, and gaps with input from relevant experts and local communities. [Stocktaking and Gap Analysis]	Understanding of existing resources and how they differ for consensus building	Identify and describe the characteristics of commonalities and gaps; established procedures and documentation; guidance on use to assist with identifying appropriate standards; connecting dataset with relevant standard
	Improve coordination and resource sharing among stakeholder organizations by compiling a diverse list of stakeholders that reflects a broad range of philosophies and frameworks on data access and sharing, and by establishing a globally accessible platform for collaboration and consensus-building. [Community Building]	Establishment of a group that reaches agreement on goals and a process of consensus building	Data being generated uses the standards; use of standards increases over time; governance structure with transparent decision-making

**Align FAIR data principles with necessary data protections.** (3)	Identify criteria for data masking—such as privacy, security, and compliance—and determine appropriate tiered access levels (e.g., public, restricted) by engaging experts across relevant sectors, including privacy, security, and regulatory compliance. [Best Practices and Standards]	Determining which data types are associated with each data masking criteria (e.g., privacy, security, compliance)	Tiered access levels identified
	Identify data types requiring protection and establish clear criteria for determining data protection needs, supported by a mechanism that connects repository developers with appropriate guidance on data protection practices. [Stocktaking and Gap Analysis]	List of data types that require data protections; development of ethical guidelines	Application of the data protections within existing data repositories

Group 6 - Incentivize researchers, database providers, and publishers to establish and adhere to best practices for data use, curation, and citation

**Milestone toward *Increased Establishing Best Practices*:**	**Strategies to reach Milestone through measure by resources (a)**	**Short-Term Outcomes (< 5 years) (b)**	**Long-Term Outcomes (+5 years) (c)**
**Incentivize participation in the creation of FAIR data.** (1)	Construct a value statement about the benefits of creating FAIR data to participation for data holders and users through the number of institutions/Projects that sign on to participate in discussions by institutional buy-in and empowering staff within institutions to have discussions with management. [Community Building]	Increased awareness among institutions and their staff regarding the importance of FAIR data; initial uptick in participation in discussions and working groups.	Institutional policy shifts toward FAIR practices; cultural shift where FAIR becomes standard in project development and data management.
	Develop ambassadors from across institutions within the biological data community to communicate with stakeholders in a range of subfields by establishing a training program for ambassadors to learn strategies to generate buy-in. [Community Building]	Ambassador training programs established; early outreach to stakeholder groups across subfields initiated.	Strong inter-institutional FAIR data advocacy network; measurable increase in buy-in and adoption of FAIR practices across subfields.

	Create functional participant groups consisting of a 60/40 split between people who have worked together previously and those that are new to the group to dive more deeply into issues that are obstacles to FAIR data production. Group should complete intensive training in team science and facilitation.<br>[Community Building]	Diverse participant groups formed and trained in team science; early identification of core FAIR data challenges.	Sustained interdisciplinary collaboration networks that break silos; concrete solutions co-developed and scaled across the data community.
	Create designations or certifications for FAIR data, similar to Open Science Framework badges through agreed upon sets of criteria for FAIR data certification, a review panel for applications for certificate, and mechanisms for monitoring, publicizing, and rewarding certificate holders.<br>[Best Practices and Standards]	Certification framework developed; pilot review panel and criteria testing initiated.	Recognition of FAIR certification as an industry standard; integration into institutional and journal assessment systems.
	Make it very easy to make data FAIR by developing training modules and curriculum to support data users and data inputters exploring the why's and how's to FAIR data, providing guidance to data creators to most appropriate tools, and providing examples and resources. Training can be uploaded to YouTube.<br>[Education and Training]	Training modules created and uploaded (e.g., to YouTube); early adopters begin to engage.	Curriculum adopted by educational institutions and organizations; a new cohort of FAIR-trained professionals.
	Appoint a communications person to research and highlight stories or case studies of questions that could not have been answered or problems that could not have been solved without FAIR data.<br>[Community Building]	Case studies and stories begin circulating via blogs, newsletters, social media.	Tangible change in stakeholder perception and recognition of FAIR data's impact; increased demand for FAIR data from decision-makers.
	Conduct targeted outreach to journals, funding agencies and data repositories to push the requirement to adhere to FAIR data principles for grant applicants and authors.<br>[Community Building]	Initial meetings and partnerships formed with key stakeholders; discussion drafts on FAIR requirements shared.	Policy shifts in journal and funding requirements; widespread enforcement of FAIR principles in publishing and grantmaking.
**Develop standards for the creation of tools that integrate data across domains.**<br>(2)	Develop a working group to understand previous 'tools' developed to see if there were any lessons learned and to agree upon standards for tool 'x'.<br>[Stocktaking and Gap Analysis]	Initial synthesis of existing tools and lessons completed; preliminary report shared.	Tool development is informed by lessons learned, preventing redundancy; harmonized approach to FAIR tooling.
	Build in flexibility to 'tool' choice to broaden alternatives and ensure integration can be applicable beyond currently existing data.<br>[Technological Capacity-Building]	Stakeholder consultation on current tool constraints and preferences.	Interoperable tools developed that support broader participation and integration with future technologies.
	Continue to revisit/update standards regularly with community feedback to ensure they are simple, dependable, and cheap.	Feedback loops established through surveys and workshops; initial revisions to standards implemented.	Continuous improvement culture sustained; standards become user-friendly and widely adopted across institutions.

	[Best Practices and Standards]		
**Develop a culture of standards for appropriate citation, attribution, and annotation mechanisms of data across domains.** (3)	Develop a variety of citation, attribution, and annotation mechanisms for rewarding proper citation and attribution, ensure annotations and data cleaning by end-users move back to the hosting collections, and make it easier to track individual people and institutions as well as their contributions in management software. [Best Practices and Standards]	Prototype tools and tracking systems created; stakeholder engagement in design phase.	Comprehensive FAIR data attribution ecosystem; academic credit and institutional recognition of contributions.
	Develop training modules covering ethics, practices, and value of proper data citation through exemplar papers that conform to best practices in data citation and attribution. [Education and Training]	Exemplar resources identified and packaged into learning modules.	Widespread integration of ethics and citation training into academic and professional development programs.
	Establish criteria for professional advancement in relevant fields to include measures of the publication/curation/management of data and information. [Best Practices and Standards]	Draft advancement criteria developed and shared with institutions and professional societies.	Data stewardship formally recognized in tenure/promotion; data professionals gain equitable standing in the research ecosystem.
	Advocate for journal editors to require appropriate acknowledgement and citation of data sources criterion be used in peer review of submitted papers and require papers that use and cite data to include the license for each record or data set. [Community Building]	Engagement with editorial boards; policy proposal drafts created.	Standardization of data citation practices in peer review; greater compliance and transparency in data usage and licensing.

BIOFAIR DATA NETWORK

Appendix F. Enhanced AI Summary

FINAL WORKSHOP:
Building the social & technological infrastructure for data integration

February 13, 2025

SUMMARY

The final workshop of the BIOFAIR Data Network Project was held virtually on February 13, 2025 (10:00 am–5:00 pm EST / 9:00 am–4:00 pm CDT / 8:00 am–3:00 pm MST / 7:00 am–2:00 pm PST). The workshop brought together an expansive configuration of stakeholders, including a subset of attendees from the listening sessions, to develop recommendations and create a roadmap towards a FAIR, open, integrated data network. This document is an augmented version of the summary generated by Zoom's AI Companion.

Overall, 75 participants joined the workshop, including 19 BIOFAIR steering committee members (Appendix A). Workshop attendees discussed the integration of biological and environmental data, focusing on technological and social aspects, challenges, and potential solutions. Participants explored various projects and initiatives aimed at improving data access, sharing, and standardization across disciplines while considering issues of equity, indigenous perspectives, and data sovereignty. The conversation ended with an exercise to develop a collaborative, actionable, adaptable, and community-informed roadmap for building a FAIR, open, and integrated biological and environmental data network

WORKSHOP SYNTHESIS

PART I: Presentations & Discussions on Technological Aspects of BIOFAIR

Introduction to the BIOFAIR Data Network

Matthew Sheik, a collections assistant at Denver Botanic Gardens and a member of the BIOFAIR Steering Committee, presented on the role of BCoN in advancing biological collections. BCoN, co-founded by AIBS, NSCA, and SPNHC, authored the Extended Specimen Network (ESN) report to enhance the integration and utility of biological collections. BCoN's latest effort, the BIOFAIR Data Network project, funded by the National Science Foundation, explores what would be needed to build an integrated, open, and FAIR biological and

environmental data network. During six virtual domain-focused listening sessions organized in the summer of 2024, stakeholders identified key challenges for building such a network, including data access, discovery, integration, standardization, ethical considerations, and training needs.

Definition of biodiversity data integration: *The process of combining various kinds of data from different sources to provide a unified view for both users and computer applications, and allow combination, manipulation, and analysis of these and new data.*

Common themes from the 6 sessions included the need for standardized metadata, FAIR data-sharing principles, improved citation practices, and sustainable infrastructure. Key recommendations included:

- Enhancing data accessibility, particularly in underserved regions.
- Establishing ethical data standards that respect indigenous knowledge.
- Incentivizing best practices for data documentation and citation.
- Increasing the availability of biological and environmental data.
- Developing common language, ontologies, data models, and persistent identifiers to improve data integration.
- Expanding training and education to bridge knowledge gaps.
- Ensuring long-term funding and infrastructure for data preservation.

The findings highlight the importance of collaboration across domains to create a comprehensive, FAIR (Findable, Accessible, Interoperable, and Reusable) biological data network.

Workshop Objectives

David Kunkel, a PhD candidate at Oklahoma State University and a member of the BIOFAIR Steering Committee, outlined the vision for the BIOFAIR network and laid out the workshop objectives:

1. Identify key individuals and organizations that need to be involved in the network and explore what the BIOFAIR network could accomplish.
2. Determine necessary actions to build the network and how these might be prioritized to accomplish short- and long-term outcomes for establishing a functional network of people and data.

Vision for a BIOFAIR Data Network: *An open consortium of biological and environmental data providers, managers, and users that would facilitate the integration of a network of data into a technological framework for use in primary research, education, and policy.*

Following these stage-setting presentations, participants heard from invited speakers.

Current data integration efforts at the Global Biodiversity Information Facility (GBIF) (Kyle Copas)

Kyle Copas, Head of Communications at GBIF, discussed data integration challenges and strategies at GBIF. He highlighted the balance between technical and sociocultural aspects of data sharing, emphasizing that impediments to data integration were largely social rather than technical. He discussed the evolution of the Darwin Core model to better serve various scientific communities. GBIF has expanded its data model to accommodate diverse use cases while simplifying data publishing methods for different research groups. Kyle also addressed community engagement, explaining GBIF's systematic approach to understanding different data-sharing networks, policy relevance, and openness to collaboration. He shared examples, including efforts to integrate health-related data through partnerships with TDR—a special program for research and training in tropical diseases co-sponsored by UNICEF, UNDP, and WHO—and targeted funding for data mobilization and publication incentives. Another focus was on ecological survey and monitoring data, with ongoing efforts to improve data accessibility and usability. Overall, GBIF continues to refine its technical infrastructure and foster collaboration to enhance biodiversity data integration worldwide.

Digital Twin and DiSSCo (Sharif Islam)

Sharif Islam, data architect for the DiSSCo project, discussed the role of digital twins in natural science collections. He explained how DiSSCo and other organizations are exploring digital specimens—digitized, machine-actionable versions of physical natural history specimens—aligned with FAIR principles. The DiSSCo project, a European Union-funded initiative, is developing the legal and technical infrastructure to unify and enhance digitized biodiversity data from museums, universities, and botanical gardens across Europe. Sharif emphasized the importance of persistent identifiers, data harmonization, community-driven annotations, and machine-assisted metadata enrichment. The goal is to improve data integration, accessibility, and citation, ensuring digital specimens can dynamically evolve alongside their physical counterparts.

A Vision for Continental-Scale Biology (Jeannine Cavender Bares)

Jeannine Cavender-Bares presented findings from a National Academies report on continental-scale biology she co-authored, emphasizing the need for integrating biology across multiple scales to address pressing global issues like climate change and biodiversity loss. Sponsored by NSF, the report highlights the fragmentation in biological sciences and seeks strategies to unify research across organizational, spatial, and temporal scales. She discussed how NSF initiatives, such as Macrosystems Biology and the Biology Integration Institutes, support this vision. Continental-scale biology involves studying biological processes and patterns that emerge at broad scales and require multi-scale integration, from molecular to biosphere levels and local to global extents. A systems approach is crucial, acknowledging human influence as integral to biological processes. The report's focus on research infrastructure underscored the role of observational networks like the National Ecological

Observatory Network (NEON), advanced tools such as remote sensing and artificial intelligence, and synthesis centers that facilitate large-scale data analysis. Emerging technologies, including hyperspectral and LiDAR data from airborne and spaceborne platforms, offer new ways to monitor ecosystem health, predict below-ground conditions, and support biodiversity conservation. Key themes of continental-scale biology include understanding biodiversity and ecosystem function, assessing resilience and vulnerability, analyzing connectivity across landscapes, and ensuring the sustainability of ecosystem services. These efforts help inform decision-making for land stewardship, conservation, and global biodiversity frameworks. Overall, the report advocates for interdisciplinary collaboration and enhanced infrastructure to better understand and manage biological systems at continental scales.

SciX - Science Explorer Digital Library Portal (Sunny Narayanan)

Sunny Narayanan presented his work as the lead ambassador for NASA's Science Explorer (SciX) program, an initiative expanding the NASA Astrophysics Data System into an interdisciplinary, open-access digital library. Launched in 2023, SciX aggregates scientific literature across various fields, including Earth science, planetary science, astrophysics, heliophysics, and, more recently, the biological sciences. The platform currently contains about 25 million publications and aims to enhance research transparency, enable cross-disciplinary comparisons, and integrate AI tools for improved data accessibility. SciX supports NASA's biological and physical sciences research, including space biology, by linking scientific literature to studies on human adaptation to space. It also fosters community engagement through advisory groups, outreach initiatives, and interactive search features.

Discussion

A brief Q&A and discussion session followed presentations from the four speakers. Key topics included the disambiguation process and the filter system in the SciX platform, accessibility and rights to open-source documents, and the importance of integrating organismal biology into large-scale ecological research. Joseph Cook raised concerns about the lack of emphasis on organismal sampling in large-scale biological research, particularly with museum collections and their role in documenting ecological change. Jeannine Cavender Bares acknowledged the importance of this issue and suggested further discussions and potential publications on the topic. The session concluded with a call for participants to contribute to a stakeholder list to identify key contacts and support future discussions on an integrated data network.

PART II: Presentations & Discussions on Social Aspects of BIOFAIR

Building a community – Collective Impact Model (Kendra Spence Cheruvelil)

Kendra Spence Cheruvelil, a professor in the Department of Fisheries and Wildlife at Michigan State University, presented on building effective science communities within interdisciplinary research teams. She shared insights from her work co-directing the Data Intensive Landscape Limnology Lab, where they transitioned from studying individual lakes to large-scale lake

ecology, building the necessary research infrastructure along the way. She emphasized that successful teams require more than just data and tools; they need diverse, interdisciplinary teams with strong communication, conflict resolution, and teamwork skills. Cheruvelil stressed the importance of co-developing team policies to address expectations, roles, and power dynamics. She also encouraged scientists to seek training in collaboration, project management, and conflict resolution to create high-performing teams and shared resources for improving team collaboration.

Building the MEPA community (Joe Cook)

Joseph Cook, curator of mammals at the Museum of Southwestern Biology, talked about building the community for the Museums and Emerging Pathogens in the Americas (MEPA) project, which integrates museum resources into emerging pathogen research across the Americas. He highlighted growing global challenges, such as biodiversity loss, climate disruption, and the rise of emerging pathogens. Cook emphasized the need for “holistic collecting” in museums, where specimens are gathered alongside related organisms and pathogens, creating a comprehensive dataset for future research. He shared how the MEPA community, developed with collaborators across the Americas, fosters collaboration in pathogen research. This network, involving over 270 participants from 16 countries, has led to joint efforts in pathogen surveillance, capacity building, and addressing issues like the Nagoya Protocol. Cook also discussed the importance of digitizing museum collections, particularly in Latin America, to provide better access to vital data. He stressed the need for sustainable, locally built infrastructure and capacity to enable effective biodiversity and pathogen research, particularly in biodiverse, resource-limited countries.

Q&A and Discussion

Participants then engaged in a discussion centered around the challenges and opportunities in scientific collaboration, policy engagement, and data integration. Concerns were raised about the barriers to accessing biological samples due to material transfer agreements, emphasizing the difficulties encountered even within a single country. Joseph Cook responded by highlighting the reluctance of scientists to engage in policy, noting that many still debate whether professional societies should be involved in policy matters. He stressed the need for greater engagement to address these barriers. Deborah Paul introduced another challenge related to institutional biohazard policies, which complicate specimen handling due to concerns over potential viruses. Joseph Cook acknowledged the issue and advised working with institutional safety boards while pointing to emerging concerns such as prions and chronic wasting disease.

Donat Agosti then shifted the discussion to taxonomy, questioning how institutions ensure consistency in species identification. Cook explained that mammal taxonomy relies on the Mammal Diversity Database and that virus taxonomy is managed by an international convention. However, he acknowledged the difficulties in integrating Latin American collections due to decentralized databases.

Mike Webster inquired about Cook's emphasis on "intentionality" in specimen collection, asking whether it referred to the collection process or subsequent usage. Cook clarified that intentionality applied to both—deciding what to collect and ensuring specimens are preserved in ways that support a broad range of research fields. He noted how curatorial responsibilities have evolved over the years, expanding from taxonomy to serving ecologists, pathogen biologists, and other researchers. He cited the University of New Mexico's early adoption of frozen tissue collections as an example of forward-thinking preservation.

Discussion: What would the BIOFAIR network enable that is not possible now?

The conversation then transitioned into a broader discussion about the potential of an integrated data network. Participants were prompted to consider what such a network could enable that is currently impossible. Stephen Formel suggested that, beyond research, the network could help develop sustainable funding mechanisms by reducing reliance on organizations like the NSF. Joseph Cook suggested that securing support from a billionaire could address funding challenges. Deborah Paul added that early warning systems could be a key application of integrated data, comparing it to Japan's seismic alert system. She pointed to wastewater surveillance as an example of a data source that could be leveraged for early warning efforts. The discussion concluded with a focus on identifying the beneficiaries of early warning systems and the importance of ensuring that these systems are effectively designed to serve relevant stakeholders. Participants emphasized that integrated data efforts should not only facilitate scientific research but also have tangible applications in policy-making, public health, and environmental monitoring.

Challenges towards building a global BIOFAIR data network (Israel Borokini)

Israel Borokini discussed the challenges of building a globally equitable biodiversity data network, focusing on disparities between the Global North and South, particularly in Africa. He highlighted that while biodiversity is richest near the equator, these regions often lack sufficient research funding, cyber infrastructure, and trained professionals. Due to limited resources, scientists in the Global South frequently self-fund research, leading to narrower studies and a reliance on external infrastructure. Borokini also critiqued the underutilization of biodiversity data in the Global South, raising concerns about "colonial science," where data from these regions is used by researchers elsewhere with little local collaboration. To address these issues, he advocated for in-country training programs, better governmental support for research and development, improved digital infrastructure, and greater engagement from diaspora scientists. He also highlighted the need for improved data integration, particularly in databases like GenBank, to facilitate research efficiency.

Indigenous perspective on data sharing (Daniel Wildcat)

Daniel Wildcat, a professor at Haskell Indian Nations University, provided an Indigenous perspective on data sharing. He emphasized that tribal nations, recognized as sovereign entities, had the right to control their data, including its collection and use. He highlighted that

tribes also functioned as public health authorities, reinforcing their sovereignty over health-related data. Wildcat stressed that data sovereignty was not just a right but also a responsibility, aligning with Indigenous traditions of ethical stewardship. He pointed out that open data posed challenges for tribal nations due to historical extractive practices in science, which often disregarded Indigenous consent and control. He referenced the UN's principle of free, prior, and informed consent as essential for FAIR data protocols. Wildcat then expanded the discussion by reframing data within an Indigenous worldview, arguing that the natural world should be seen not as a collection of resources but as a network of relatives. He advocated for a shift from focusing on inalienable rights to embracing unalienable responsibilities, emphasizing relational accountability over individual entitlement. He concluded by urging the use of technology to enhance life systems rather than prioritizing human convenience and profit. He called for a perspective rooted in "ancestral intelligence" or AI 1.0, integrating Indigenous knowledge into modern scientific and technological frameworks to foster sustainability and ethical governance.

Discussion on the Role of Responsibility in Open Data and Scientific Collaboration

A brief discussion followed, covering several key topics, including the challenges of engaging African leaders in supporting African scientific research, the need for local funding rather than reliance on the Global North, and the role of professional societies in advocating for research support. There was also a focus on improving data management training within universities and institutions in the Global South, including in Africa, to enhance accessibility and scientific contributions. Additionally, the conversation highlighted the importance of researchers having identifiers (such as ORCID) to facilitate collaboration and recognition. Another major theme was the ethical concerns surrounding open data and artificial intelligence (AI), particularly regarding responsibility, privacy, and potential misuse of information. Daniel Wildcat emphasized the need for discussions on AI that consider ethical responsibilities alongside rights, referencing his books *Red Alert!: Saving the Planet with Indigenous Knowledge* and *On Indigenuity: Learning the Lessons of Mother Earth* for further insights.

Part III: Developing the BIOFAIR Data Network Roadmap

Introduction to the Exercise

Cameron Pittman and Matthew Sheik introduced a roadmapping exercise focused on identifying strategies to achieve key milestones for improving biological and environmental data findability, accessibility, interoperability, and reusability. Participants were divided into 6 breakout groups, based on the six needs identified in the Listening Session Summary report, where they discussed strategies, short- and long-term outcomes, progress measurement, and needed resources to address those needs.

Identified Needs

1. Enhance the availability of biological and environmental data to support research and decision-making.
2. Improve capacity for data integration
3. Establish sustainable funding models and streamlined infrastructure to ensure the long-term preservation, accessibility, and usability of key biological and environmental data resources.
4. Build a robust training infrastructure to equip researchers, data managers, and early career scientists with the skills needed for the effective use, sharing, and maintenance of biological and environmental datasets.
5. Develop mechanisms for addressing inequities in data access and policy to ensure the ethical use of data.
6. Incentivize researchers, database providers, and publishers to establish best practices for data use, curation, and citation.

Roadmap components included milestones, strategies, short-term and long-term outcomes, measures of progress, and resources needed.

Roadmap components and definitions:

- **Milestone**: A specific point along the journey to achieving a goal can act as a checkpoint to help track progress.
- **Strategies**: A plan of action designed to achieve a long-term or overall aim, and can be broad in scope and encompass the final achievement.
- **Measures**: Ways in which the action will be measured. These could be potential outputs or set goalposts.
- **Outcomes**:
    - Short-Term: Outcomes achievable within 5 years after implementation of the resources and actions.
    - Long-Term: Outcomes achievable after 5+ years.
- **Resources**: These qualify as anything that is needed to achieve the respective SMART Goal. Examples include infrastructure, personnel, databases, funding, etc.

The session aimed to flesh out a structured roadmap with actionable steps, which would be consolidated, refined, and converted into a visual graphic for the final report. Facilitators guided discussions within each breakout, and the findings were then summarized in a report-out session.

Breakout Session Findings and Summaries

Following the breakouts, participants reconvened to discuss their findings. The groups focused on strategies to improve biological and environmental data availability, integration, and

sustainability. Key topics included expanding data types, fostering collaboration and data sharing, enhancing interoperability, and securing long-term funding.

Group One emphasized dedicated funding, standardized data repositories, and better attribution mechanisms. They discussed the need for expanding the availability of data types, including acoustic data, observational data, and small focus data sets. He also highlighted the importance of creating a protocol for direct data upload and a repository for publications.

Group Two discussed the importance of persistent identifiers, common ontologies, and integrating APIs to bridge data silos. They shared the group's discussion on improving the established culture of collaborative and open data sharing, emphasizing the development of accreditation and attribution mechanisms.

Group Three highlighted the importance of investing in human capital for cyber infrastructure maintenance and development, creating resilient funding models beyond government sources, and the need for a centralized resource for training.

Group Four addressed training infrastructure, developing and integrating curriculum resources, advocating for bottom-up skill-sharing, flexible educational resources, and defining clear and achievable career competencies.

Group Five discussed the need for developing guidance for equitable access to data and technologies and the importance of coordinating among different stakeholder communities.

Group Six highlighted the need for incentivizing participation in the creation of FAIR data, developing standards for the creation of tools that integrate data across domains, and developing a culture of standards for appropriate citation, attribution, and annotation mechanisms.

Overall, the discussion underscored the need for sustainable data management, collaboration, and accessibility across disciplines.

Part IV: Conclusions and Next Steps

Genomic Adaptation and Resilience to Climate Change - GenARCC (James Macklin)

The concluding presentation by James Macklin highlighted the *Genomic Adaptation and Resilience to Climate Change (GenARCC)* project, a large-scale Canadian initiative integrating genomics, ecological, and climate data to address climate change impacts on biodiversity, ecosystems, food security, and health. The project, involving multiple federal agencies and universities, aims to assess species adaptation, monitor environmental changes, and predict future vulnerabilities. Macklin emphasized the role of genomic tools, data integration, and computational power in analyzing adaptation and mitigation strategies. Key aspects include leveraging existing data, studying microbiomes, monitoring emerging pathogens, and ensuring

open, standardized data for broad accessibility. The talk underscored the importance of interdisciplinary collaboration, high-performance computing, and standardized climate datasets for effective research and policy-making.

Following the presentation, the discussion focused on the importance of integrating communication experts into research projects, not just as an end goal but as an ongoing engagement opportunity. Hiring communicators needs to be seen as a way to involve them in meaningful dialogue rather than simply an endpoint of knowledge dissemination. James Macklin shared an example of working with an Advisory Board member specializing in sociology and forestry, a collaboration that helped them recognize the significance of social engagement in research. The discussion highlights that research projects, often publicly funded, aim for practical application, making communication and engagement essential components.

Next Steps

The concluding discussion focused on identifying next steps to sustain progress on the strategies identified during the roadmapping exercise to build an integrated, open, and FAIR data network. The discussion emphasized forming working groups to address key needs identified in the roadmap, coordinate efforts, and seek funding opportunities. Potential avenues for supporting future activities include the working group mechanism available from the Environmental Data Science Innovation and Inclusion Lab (ESIIL) at the University of Colorado at Boulder, NSF programs like FAIROS and Midscale Research Infrastructure, and other funding opportunities. Participants highlighted the importance of prioritizing actionable initiatives, such as improving access to persistent identifiers in under-resourced communities. International collaboration was also recognized as a crucial but challenging aspect requiring sustainable funding.
